# Supplementary material for: Functional annotation of regulatory elements in rainbow trout uncovers roles of the epigenome in genetic selection and genome evolution
Source: Gigascience. 2024 Dec 4;13:giae092. doi: 10.1093/gigascience/giae092 (PMC11629980; doi:10.1093/gigascience/giae092)
Supplement: giae092_Supplementary_Files [file giae092_supplementary_files.zip › Additional File 2.pdf]

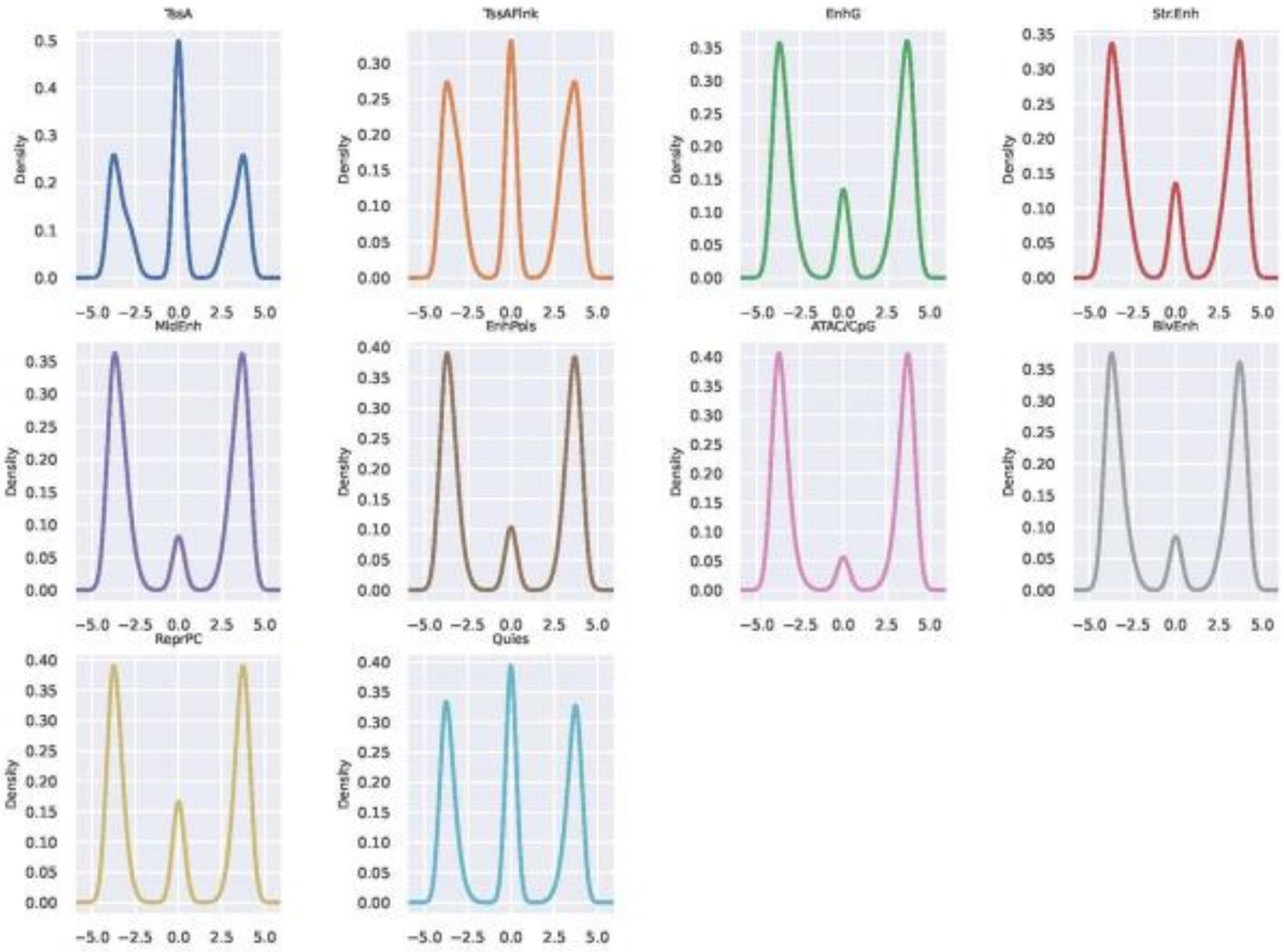

Additional file 2. Density of each chromatin state relative to the position of TSS of the protein-coding genes

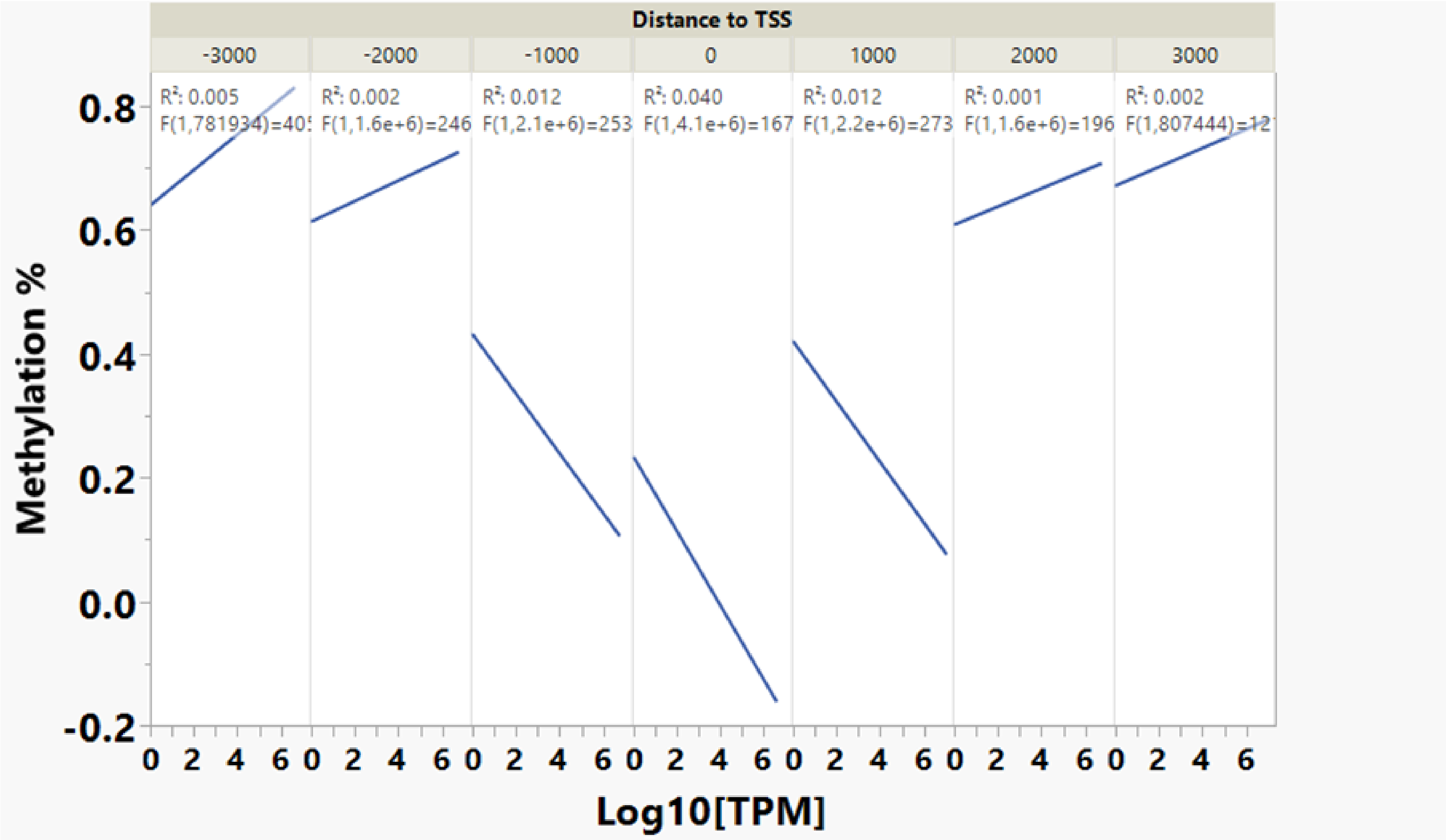

Additional file 2. Weak correlation between DNA methylation and gene expression, negative within ±1 kb flanking TSS and positive elsewhere
