# Supplementary material for: Functional annotation of regulatory elements in rainbow trout uncovers roles of the epigenome in genetic selection and genome evolution
Source: Gigascience. 2024 Dec 4;13:giae092. doi: 10.1093/gigascience/giae092 (PMC11629980; doi:10.1093/gigascience/giae092)
Supplement: giae092_GIGA-D-24-00104_Revision_2 [file giae092_giga-d-24-00104_revision_2.pdf]

GigaScience

Functional annotation of regulatory elements in rainbow trout uncovers roles of the epigenome in genetic selection and genome evolution

--Manuscript Draft--

Manuscript Number:	GIGA-D-24-00104R2
Full Title:	Functional annotation of regulatory elements in rainbow trout uncovers roles of the epigenome in genetic selection and genome evolution
Article Type:	Research
Funding Information:	National Institute of Food and Agriculture (2020-67015-30770) — Professor Mohamed Salem<br>National Institute of Food and Agriculture (2023-67015-39742) — Professor Mohamed Salem<br>National Institute of Food and Agriculture (2021-67015-33388) — Professor Mohamed Salem
Abstract:	Rainbow trout (RBT) has gained widespread attention as a biological model across various fields and has been rapidly adopted for aquaculture and recreational purposes on six continents.<br>Despite significant efforts to develop genome sequences for RBT, the functional genomic basis of RBT's environmental, phenotypic, and evolutionary variations still requires epigenome reference annotations.<br>This study has produced a comprehensive catalog and epigenome annotation tracks of RBT, detecting gene regulatory elements, including chromatin histone modifications, chromatin accessibility, and DNA methylation. By integrating ChIP-seq, ATAC-seq, Methyl Mini-seq, and RNA-seq data, this new regulatory element catalog has helped to characterize the epigenome dynamics and its correlation with gene expression. The study has also identified potential causal variants and transcription factors regulating complex domestication phenotypic traits. This research also provides valuable insights into the epigenome's role in gene evolution and the mechanism of duplicate gene retention 100 million years after RBT whole-genome duplication and during re-diploidization. The newly developed epigenome annotation maps are among the first in fish and are expected to enhance the accuracy and efficiency of genomic studies and applications, including genome-wide association studies, causative variation identification, and genomic selection in RBT and fish comparative genomics.
Corresponding Author:	Mohamed Salem, PhD<br>University of Maryland at College Park: University of Maryland<br>College Park, Maryland UNITED STATES
Corresponding Author Secondary Information:	
Corresponding Author's Institution:	University of Maryland at College Park: University of Maryland
Corresponding Author's Secondary Institution:	
First Author:	Mohamed Salem, PhD
First Author Secondary Information:	
Order of Authors:	Mohamed Salem, PhD<br>Rafet Al-Tobasei<br>Ali Ali<br>Liqi An<br>Ying Wang<br>Xuechen Bai<br>Ye Bi

Powered by Editorial Manager® and ProduXion Manager® from Aries Systems Corporation

	Huaijun Zhou
**Order of Authors Secondary Information:**	
**Response to Reviewers:**	Please see the attached "Response to Reviewer Comments" file on the last pages below. We attached the file to maintain the enclosed table formats.<br><br>Thank you,
**Additional Information:**	
**Question**	**Response**
Are you submitting this manuscript to a special series or article collection?	No
**Experimental design and statistics**<br><br>Full details of the experimental design and statistical methods used should be given in the Methods section, as detailed in our Minimum Standards Reporting Checklist. Information essential to interpreting the data presented should be made available in the figure legends.<br><br>Have you included all the information requested in your manuscript?	Yes
**Resources**<br><br>A description of all resources used, including antibodies, cell lines, animals and software tools, with enough information to allow them to be uniquely identified, should be included in the Methods section. Authors are strongly encouraged to cite Research Resource Identifiers (RRIDs) for antibodies, model organisms and tools, where possible.<br><br>Have you included the information requested as detailed in our Minimum Standards Reporting Checklist?	Yes
**Availability of data and materials**<br><br>All datasets and code on which the conclusions of the paper rely must be either included in your submission or deposited in publicly available repositories	Yes

Powered by Editorial Manager® and ProduXion Manager® from Aries Systems Corporation

(where available and ethically appropriate), referencing such data using a unique identifier in the references and in the “Availability of Data and Materials” section of your manuscript.<br><br>Have you have met the above requirement as detailed in our Minimum Standards Reporting Checklist?	

Powered by Editorial Manager® and ProduXion Manager® from Aries Systems Corporation

Manuscript Click here to access/download;Manuscript;R2-MS_Oct 25.docx

Functional annotation of regulatory elements in rainbow trout uncovers roles of the epigenome in genetic selection and genome evolution

Mohamed Salem[1], Rafet Al-Tobasei[2], and Ali Ali[1], Liqi An[3], Ying Wang[3], Xuechen Bai[3], Ye Bi[3], Huaijun Zhou[3]

[1]Department of Animal and Avian Sciences, University of Maryland, College Park, MD 20742-231, USA

[2]Computational Science Program, Middle Tennessee State University, Murfreesboro, TN 37132, USA

[3]Department of Animal Science, University of California, Davis, Davis, CA 95616, USA

Mohamed Salem [0000-0003-2142-6716]; Rafet Al-Tobasei [0000-0002-0578-0734]; Ali Ali [0000-0002-7058-5150]; Liqi An [0009-0008-2484-9355]; Ying Wang [0000-0001-6920-8692]; Xuechen Bai; Ye Bi; Huaijun Zhou [0000-0001-6023-9521].

ABSTRACT

Rainbow trout (RBT) has gained widespread attention as a biological model across various fields and has been rapidly adopted for aquaculture and recreational purposes on six continents. Despite significant efforts to develop genome sequences for RBT, the functional genomic basis of RBT's environmental, phenotypic, and evolutionary variations still requires epigenome reference annotations.

This study has produced a comprehensive catalog and epigenome annotation tracks of RBT, detecting gene regulatory elements, including chromatin histone modifications, chromatin accessibility, and DNA methylation. By integrating ChIP-seq, ATAC-seq, Methyl Mini-seq, and RNA-seq data, this new regulatory element catalog has helped to characterize the epigenome dynamics and its correlation with gene expression. The study has also identified potential causal

variants and transcription factors regulating complex domestication phenotypic traits. This research also provides valuable insights into the epigenome's role in gene evolution and the mechanism of duplicate gene retention 100 million years after RBT whole-genome duplication and during re-diploidization. The newly developed epigenome annotation maps are among the first in fish and are expected to enhance the accuracy and efficiency of genomic studies and applications, including genome-wide association studies, causative variation identification, and genomic selection in RBT and fish comparative genomics.

INTRODUCTION

Rainbow trout (RBT) is among the most intensively studied fish in many research areas[1]. RBT, native to North America and Asia's Pacific Ocean, has been introduced to every state and province in North America and worldwide to every continent except Antarctica. In the US, RBT is the most cultivated cool and cold freshwater fish[2]. Considerable biological knowledge has been developed for this species due to the RBT's widespread use as a model and cultivation as a food and sport fish. A plethora of knowledge is available for the biology of RBT and it serves as a complementary research model for economically important fish other than RBT, such as Atlantic and Pacific salmon species[1].

The recent decade's considerable accumulation of genomic resources underscores the escalating requirement to employ genomic methodologies in RBT-focused research and applications in aquaculture and fisheries[3]. For example, RBT is an ideal model for delving into gene and genome evolution. Its status as a partially tetraploid organism, marked by a unique whole-genome duplication event (salmonid-specific 4th WGD), with subsequent partial re-diploidization and significant genome rearrangements, is an appealing subject for genetic exploration. In addition, the potential of elevating aquaculture species, such as RBT, through genomic methodologies is critical to making superior germplasm with enhanced economic traits[3].

The availability of genome sequence references is essential for genomics-based selection. An accurately assembled and annotated genome sequence is the cornerstone, facilitating in-silico mapping and validation of SNP variants. This, in turn, streamlines the design of SNP chip assays, optimizing the precision of genetic analyses. Furthermore, the genome sequence facilitates functional genomics and proteomic approaches in RBT research[3], unraveling the intricacies of an overly complex and

duplicated genomic landscape. This approach drives advancements in genetic understanding and lays the foundation for robust genomic analyses and improvement of the RBT.

Efforts to make a pangenome reference available for RBT have begun, and at least three chromosome-level genome assemblies are now available[4, 5]. However, epigenome reference annotations for RBT are lacking and needed to understand the functional genomic basis of the rapidly domesticating RBT's phenotypic, environmental, and evolutional variations. Annotating the genome for chromatin histone modifications and accessibility is essential for identifying the genome regulatory elements. The chromatin organization of genomic regions involved in functional/regulatory interactions is more accessible to nucleases and other DNA modifying enzymes due to altered structure and binding of transcription factors[6].

Epigenetics is vital in understanding the cellular and molecular processes, including cell-type specific regulation of gene expression, cellular differentiation, genomic imprinting, embryonic development, and chromosome inactivation. Regions of open chromatin identified by ATAC-seq, combined with expression analysis, allow for associating functional/regulatory elements with transcribed genes [7-9]. Although genomic DNA sequence is mainly identical in all cells, the chromatin context of the DNA changes from tissue to tissue. Some of the most significant differences are due to post-translational histone modifications.

The ENCODE project has assayed more than a dozen different histone modifications. H3K4 methylation was first discovered in the RBT testis by Honda et al., 1975 [10]. A high abundance of H3K4me3 correlates with promoters of active genes and transcription start sites[11-14], while increased levels of H3K27me3, a repressive mark, are associated with promoters of inactive genes [15, 16]. H3K27ac is a chromatin mark of active regulatory elements and may differentiate active enhancers and promoters from their inactive counterparts [16]. H3K4me1 is a chromatin mark of regulatory elements correlated with enhancers and other distal elements but is also enriched downstream of TSS[16]. Elevated levels of H3K27ac and H3K4me1 are linked with enhancer regions and correlate with open chromatin sites[13, 17]. The combinatorial profile of these different epigenetic marks has been used to predict chromatin states in several species[18-23], including livestock. Using the profiles of histone marks in concert with open chromatin and transcription profiles allows an unprecedented view of the functional elements present in the RBT genome, which is the first in aquaculture species and among the first in fish.

DNA methylation is one of eukaryotes' major epigenetic/epigenomic mechanisms that modify the primary genetic code by converting cytosine into 5-methylcytosines (5mCs). However, in fish, large-scale gene expression studies that reveal the role of DNA methylation have been done in a few species[24-26]. Integrating the DNA methylation data with chromatin modification and accessibility can help understand the regulation of gene expression, tissue complexity, organismal development, and evolution at the systems biology level. Besides, it provides valuable molecular information for the genetic improvement of fish for food production and biomedical purposes.

The Functional Annotation of Animal Genomes (FAANG) Consortium provided functional annotations atlas of farm animal genomes, including pig, cattle, and chicken, for the first time [19, 27, 28]. Currently, there is a dearth of functional annotations for fish especially aquaculture species. Moreover, epigenomic tracks have only been comprehensively established for zebrafish [29]. In the EU, the AQUA-FAANG project aims to provide functional annotation tracks of six aquaculture species [30]. On the other hand, in the US, over the last ten years of the FAANG project, aquaculture was represented by one species, the RBT. As part of this FAANG consortium, the main aim of this study was to annotate the RBT genome for chromatin histone modifications, chromatin accessibility, and DNA methylation by integrating data from ChIP-seq, ATAC-seq, and Methyl Mini-seq together with gene expression data from RNA-seq across various tissues of the RBT. The study provides a unique RBT catalog/genome annotation tracks of several tissues in correlation with variation in gene expression. The study also reveals epigenetic functions of previously identified QTL for complex phenotypic traits important for domestication by mapping QTL onto genome tracks of the new gene regulatory elements, including promoters, enhancers, super enhancers and transcription factor binding sites. The study also offers insights into the epigenome's role in gene evolution after the genome duplication in RBT.

RESULTS

OVERVIEW OF THE SEQUENCING DATASET

Approximately 1.59 billion ChIP-seq reads, 1.06 billion ATAC-seq, 0.53 billion RNA-Seq, and 1.0 billion Methyl Mini-seq were used in these analyses, with average mapping rates of 97%, 94%, 81.3%, and 79%, respectively (Additional file 1). A total of 421,240; 1,057,603; 758,037; 1,392,453; and 1,628,755 peaks were obtained for H3K4me3, H3K4me1, H3K27ac, H3K27me3,

and ATAC, with average peak size of 749; 438; 604; 585, and 691 bp, respectively (Additional file 1).

Figure 1A shows the signal intensity of each epigenetic mark relative to the transcription start site (TSS) of the protein-coding genes. The ATAC-Seq signal peaked around the TSS. The major peaks for H3K4me3 and H3K27ac were observed at about 500 nt in front of TSS, with minor peaks shortly after TSS. H3Kme1 showed moderate peaks about 1000 nt upstream of TSS and right after.

IDENTIFICATION AND CHARACTERIZATION OF 10 CHROMATIN STATES IN THE RAINBOW TROUT GENOME

Genome-wide epigenomics mappings were generated by integrating four histone modifications ChIP-seq data sets (H3K4me3, H3K4me1, H3K27ac, and H3K27me3), chromatin accessibility (ATAC-seq) and DNA methylation (Methyl Mini-seq). Data from 6 major tissues (brain, liver, spleen, white muscle, intestine, and kidney) were included in all analyses except for ATAC-seq, where data from the first 3 tissues were available. The epigenomic marker integration predicted ten categories of chromatin states in the RBT genome (Figure 1B-F).

The first predicted two states were (1) active TSS (TssA), indicating active promoters, and (2) flanking active TSS (TssAFlnk), together covering 1.42% of the genome. Strong epigenomic signals of H3K4me3, H3K27ac, and intermediate H3K4me1 signal, with no H3K27me3, characterized these two active chromatin states. TssA has higher ATAC-seq signals compared to the TssAFlnk state. As expected, these active promoter states were enriched around protein-coding gene TSS and TSS flanking regions (2kb), Zink finger transaction factors, and highly transcribed (TPM>2) genes but depleted in the repressed genes (TPM<0.2) (Figure 1B-F).

Chromatin states three to six are composed of four types of enhancers: (1) genic enhances (EnhG) characterized by very strong open chromatin signal and moderate H3K27ac and H3K4me1 signals; (2) strong active enhancers (Str.Enh) characterized by strong H3K27ac, and H3K4me1 signals but moderate open chromatin; (3) intermediate active enhancers (MidEnh) with moderate/strong H3K4me1 signal, and (4) poised enhancers (EnhPois). It is important to note that the EnhPois showed minimal chromatin modification and openness signals, yet ChromHMM identified them as a chromatin state. The first three active enhancer states (EnhG, Str.Enh, and MidEnh) cover 3.86%, while the EnhPois spans 7.2% of the genome. These enhancers were

enriched in QTL (discussed below), highly expressed genes, the 3'UTR/TES (especially EnhPois), and gene bodies but depleted in the repressed genes (Figure 1B-F).

The seventh chromatin emission state, covering 7.25% of the genome, was characterized by relatively strong ATAC-seq signals, enrichment in CpG island regions, and moderate enrichment in the suppressed genes. The eighth chromatin state, named bivalent enhancers (BivEnh), is characterized by open chromatin (ATAC-Seq), strong repressor H3K27me3 signal, and weak promoter/enhancer signals from H3K4me3, H3K27ac, and H3K4me1. The ninth chromatin state represented the repressed/polycomb (ReprPC) regions spanning 2.57% of the genome and moderately enriched in 3'UTR/TES (Figure 1B-F). Both BivEnh and ReprPC were enriched in CpG islands and genes with no or minimal expression. The tenth chromatin status was quiescent (Quies), with poor chromatin modification signals covering most of the genome (77.67%) (Figure 1B-F).

The chromatin states were used to generate genome annotation tracks available through the UCSC genome browser (see data availability). Table 1 summarizes the annotation tracks characterization with 515,159 chromatin stats; the active chromatin states (1-5) represent 24.8% of the state counts, and the non-active states (6-10) represent 75.2%. There were 47,433 active promoters, 80,404 active enhancers (EnhG, Str.Enh, and MidEnh), and 50,353 repressed enhancers (EnhPois and BivEnh). Table 1 also shows each chromatin state's mean and median length, with the enhancers' medians ranging between 400 and 1000 bp and a repressed polycomb median of 2000 bp.

Figure 1G shows an example of the UCSC genome browser tracks displaying the chromatin regulatory states at the Myosin light chain 1 (MLC1) gene in 6 tissues. Only in muscle is MLC1 flanked by strong enhancers and active TSS states; the other five tissues showed poised enhancers or quiescent states.

The density of each chromatin state relative to the position of TSS of the protein-coding genes is shown in Additional file 2. The TssA and, to a lesser extent, TssAFlnk, showed maximum enrichments at TSS. The other chromatin states showed enrichment around 5 kb on both sides of TSS.

The ATAC-seq data was missing in three tissues. The ChromHMM tool was found to be reliable for predicting states based on combinations of available chromatin tracks. In a study using the mouse ENCODE project data, ChromHMM accurately assigned chromatin states even when certain marks, such as ATAC-seq, were missing [31]. The research concluded that the absence of certain marks did not significantly impact the assignment. Although the states most affected were those most enriched in the missing mark in a ten-mark model, the opposite was not necessarily true, highlighting redundancy between the marks.

To address the effect of the missing ATAC-seq data in three tissues, we created a separate 10-state chromatin model without ATAC-seq data and uploaded the bed files to the journal portal *Folder name:* TroutChromHMm_State_without_ATAC_seq). A quick comparison of enhancers with and without ATAC-seq data revealed a 25% reduction in genetic enhancers and a 23% increase in strong enhancers using the muscle and kidney data. The mouse ENCODE paper reported similar differences in the number of enhancers [31]

DNA METHYLATION RELATIVE TO THE CHROMATIN STATES

There were distinct patterns of DNA methylation near and within each chromatin state, as shown in Figure 1H. All the active chromatin states (1-5) were hypomethylated compared to their flanking regions. As expected, the promoter TssA and its flanking regions TssAFlnk were strongly hypomethylated. Similarly, all the active enhancers (EnhG, Str.Enh, MidEnh) were moderately hypomethylated.

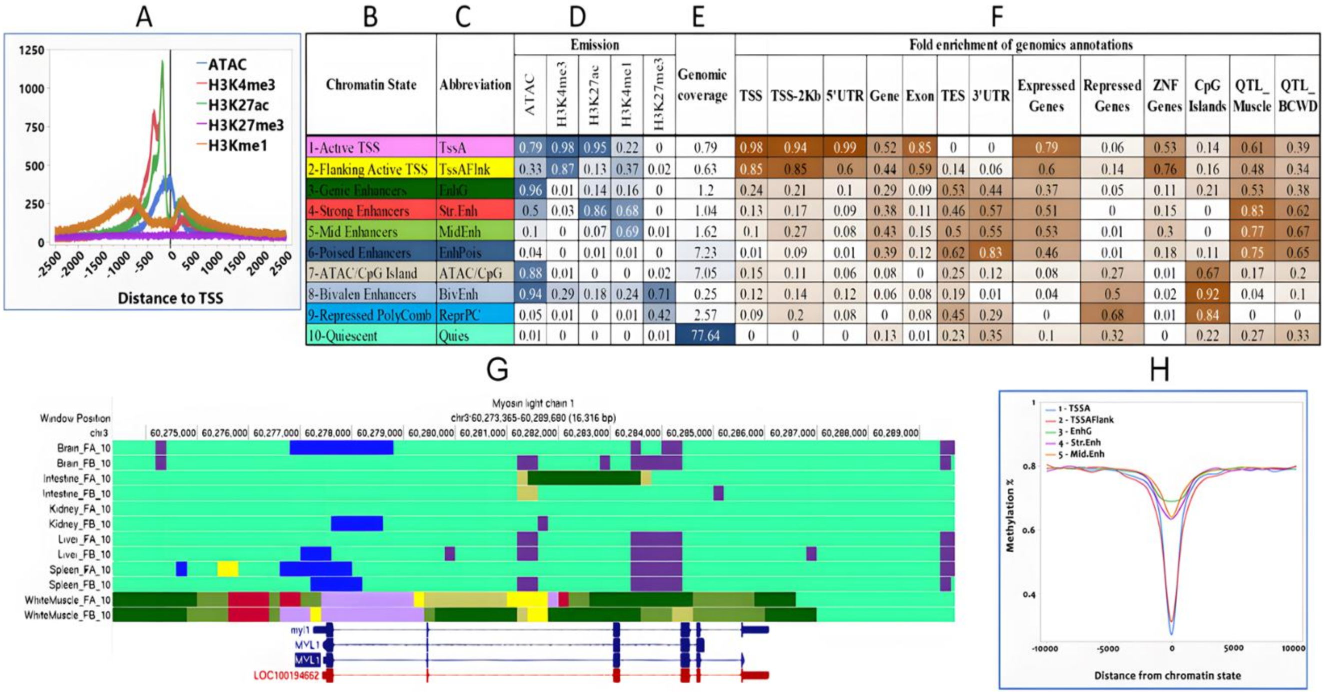

Figure 1. Discovery and characterization of chromatin marks and states in the rainbow trout genome. (A) Epigenetic mark's signal intensity from ATAC-Seq, H3K4ME3, H3K27as, H3Kme1, and H3K27me3 ChIP-Seq relative to the protein-coding genes' TSS. **(B, C)** Names and abbreviations of 10 chromatin states identified in the rainbow trout genome. **(D)** Epigenetic mark probabilities associated with each chromatin state indicated in numbers (0-1) and color intensity. **(E)** Percentage of genomic coverage of each chromatin state. (**F**) Enrichment of each chromatin state associated with various genomic annotations, including genes, TSS and flanking regions(±2 kb around TSS and TES), expressed genes (TPM ≥ 2), and repressed genes (TPM < 0.2), CpG islands, and QTL for fish/muscle growth, fillet quality and bacterial cold water disease (BCWD). **(G)** UCSC genome browser tracks showing the landscape of the chromatin states at the MLC1 gene in 6 tissues. Only in muscle is MLC1 flanked by strong enhancers (Red), weak enhancers (Dark green) and active TSS states (purple). In the other tissues, MLC1 had quiescent (light green) or poised enhancers (blue). **(H)** Average methylation levels relative to the position of each active chromatin state (1-5).

Table 1. Count, percentage, and mean/median length (bp) of each chromatin state.

State	Count	Percentage	Mean/Median state length (bp)
**1-TSSA**	496,173	4.33	1382/1200
**2-TSSAFlnk**	604,309	5.27	1081/1000
**3-EnhG**	1,167,656	10.19	1008/600

**4-Str.Enh**	651,095	5.68	1550/1000
**5-MidEhh**	1,439,241	12.56	993/600
**6-EnhPois**	1,520,923	13.27	6295/3800
**7-ATAC-CpG**	2,962,192	25.85	1078/400
**8-BivEnh**	254,795	2.22	574/400
**9-ReprPC**	618,191	5.39	4762/2000
**10-Quies**	1,746,493	15.24	179332/88600
**Total**	11,461,068	100.00	

HISTONE MODIFICATION ASSOCIATION WITH GENE EXPRESSION

Histone Marks Correlation with Gene Expression

We characterized the enrichment of the tissue-specific histone marks at promoter regions of tissue-specific expressed genes among six tissues. To do that, genes showing more than 10-fold increases in expression compared to the rest of the tissues or more than 1 TPM value with zero TPM expression in other tissues were first identified. Then, histone marks uniquely identified within ±3 kb from TSS (including -3kb of the promoter region) of the same gene showing tissue-specific expression were cross-listed. The number of the tissue-specific histone marks was divided by the total number of each histone mark in the genome to obtain a normalized relative abundance of each histone mark. Data showed that H3K4me1 was enriched in the tissue-specific genes compared to the same genes in other tissues where the genes are silent or scarce (Chi-square P-value <0.001). Conversely, H3K27me3 was enriched in the silenced genes, compared to the tissue-specific expressed genes (Chi-square P-value <0.001, Figure 2A, Additional file 1).

We also looked at the association of histone marks within ±3kb of TSS to gene expression. Densities of chromatin marks ATAC-Seq, H3K4me1, H3K4me3, and H27Kac were higher in the genes with expression values more than 1 TPM (log10 TMP equals zero). On the other hand, H3K27me3 chromatin mark density was higher in genes with less expression (Figure 2B, Additional file 3). There was significant correlation between the histone marks and the Log10 TPM values (P-value <0.001, R^2=0.074).

Chromatin States Correlation with Gene Expression

We identified 5,551 tissue-specific chromatin states within ±10 Kb of genes' TSS. There were 2,150 genes with tissue-specific gene expression and chromatin states, suggesting a correlation in

gene expression (Additional file 1). All the active chromatin states (states 1-5, active promoter, and enhancers) were enriched in genes with tissue-specific expression, especially the strong enhancers. Notably, EnhPois and, to a lesser extent, ATAC-CpG states were also enriched, indicating the involvement of other epigenetic mechanisms in regulating gene expression (Figure 2C). To get more insight into the correlation between chromatin state and gene expression, we looked at the distribution of each chromatin state density near genes with various relative gene expression levels. As seen in Figure 2D, the chromatin state densities of the open chromatin states within ±3kb of TSS, including TssA and TssAFlnk, and enhancers, including EnhG, Str.Enh, MidEnh, and EnhPois were higher in the genes with expression than 1 TPM (log10 TMP equals zero). On the other hand, chromatin states RepPC, the ATAC-CpG, and BivEnh did not show characteristic density patterns relative to gene expression. There was negligible correlation between the chromatin states and the Log10 TPM values ($R^2<0.01$), though.

DNA METHYLATION CORRELATION WITH GENE EXPRESSION

We characterized the methylation level near and within genes, ±10 kb flanking TSS. The mean level of CpGs methylation more than ±5 kb flanking TSS was about 75%; however, a sharp decrease in DNA methylation to about 10% on average was observed at the TSS. (Figure 2E). Regarding the DNA methylation correlation with gene expression, our data showed a weak (R^2= 0.002-0.04 depending on the distance to TSS) but statistically significant correlation between the average percentage of DNA methylation within ±3 kb flanking TSS and gene transcription expression (p-value < 0.0001) (Additional file 2). As seen in Figure 2F, there was a trend of negative correlation between DNA methylation and gene expression, especially of the most highly

expressed genes, with a long10 TPM value of more than 3; the correlation varies between tissues, though.

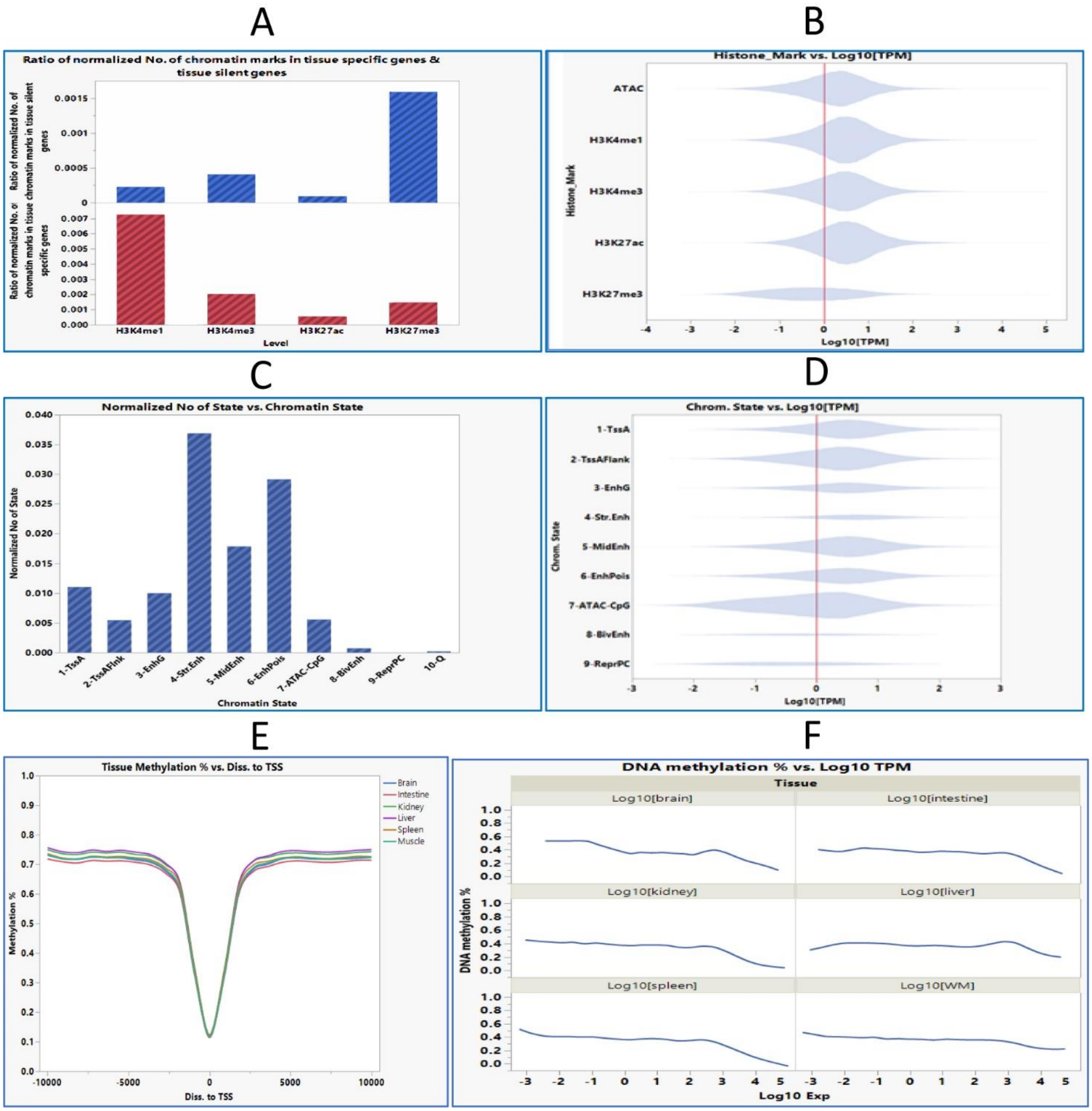

Figure 2. Histone modification and chromatin state correlation with gene expression **(A)** Enrichment of H3K4me1 histone mark within ±3kb of TSS of the tissue-specific genes (top) and H3K27me3 in the genes silenced in other tissues (bottom). **(B)** Densities of the chromatin marks within ±3kb of TSS relative to gene expression. ATAC-Seq, H3K4me1, H3K4me3, and H27Kac density were higher in the genes with expression levels of more than 1 TPM (log10 TMP equals zero). Conversely, H3K27me3 density was higher in genes with less expression. **(C)** Enrichment of chromatin states (1-7), particularly Str.Enh within ±3kb of TSS of the tissue-specific

genes. **(D)** Densities of the open chromatin states within ±3kb to TSS relative to gene expression. Chromatin state densities of TssA and TssAFlnk and enhancers EnhG, Str.Enh, MidEnh, and EnhPois were higher in the genes with expression than 1 TPM (log10 TMP equals zero). Conversely, chromatin states RepPC and the ATAC-CpG and BivEnh did not show characteristic density patterns relative to gene expression. **(E)** Average methylation percentage relative to TSS. **(F)** DNA methylation percentage relative to gene expression (log10 TMP)

DETECTION AND CHARACTERIZATION OF SUPER-ENHANCERS

We identified a total of 5,799 nonredundant super-enhancers (SE) in all studied tissues (Additional file 4). Super enhancers are clusters of enhancers enriched within 12.5 Kb of the genome. Figure 3A shows the ranked SE identified by HOMER based on an extremely high H3K27ac signal compared to conventional enhancers[32]. There was 5,104 SE within or neighboring 4,120 genes within 10Kb. Of those SE, there was an average of 850.5 SE in all tissues, ranging from 630 in the spleen to 1,167 in the intestine (Figure 3B, Additional file 4). The SE had an average length of 25,234bp, reaching a maximum length of 133Kb (Figure 3C). Figure 3D shows the chromosome distribution of the SE with an average of 159 SE per chromosome. The SE were generally shared between tissues, with 599 (13.8%) SE ubiquitously existing in all tissues and only 805 (10.3%) SE exiting in a single tissue. For example, a muscle-specific SE at location NC_048582.1:54022672-54039559 was associated with the muscle-specific gene Guanosine Monophosphate Reductase (GMPR). The SE were enriched around the gene TSS (Figure 3E). SE were also enriched in highly expressed genes with 4,737 unique SE overlapping with expressed genes (TPM values > 2) and only 286 SE overlapping in the repressed genes (TPM<0.2). Figure 3F shows an example of a super enhancer with H3K27ac signal flanking the C1QTNF4 gene only in muscle compared to a typical enhancer in all other tissues. GO enrichment analysis of the SE neighboring genes showed involvement in important molecular functions, including catalytic activity, DNA, and metal/ion binding. In the biological process, SE genes were enriched in biosynthetic, cellular metabolic process, and transcription (Additional file 4).

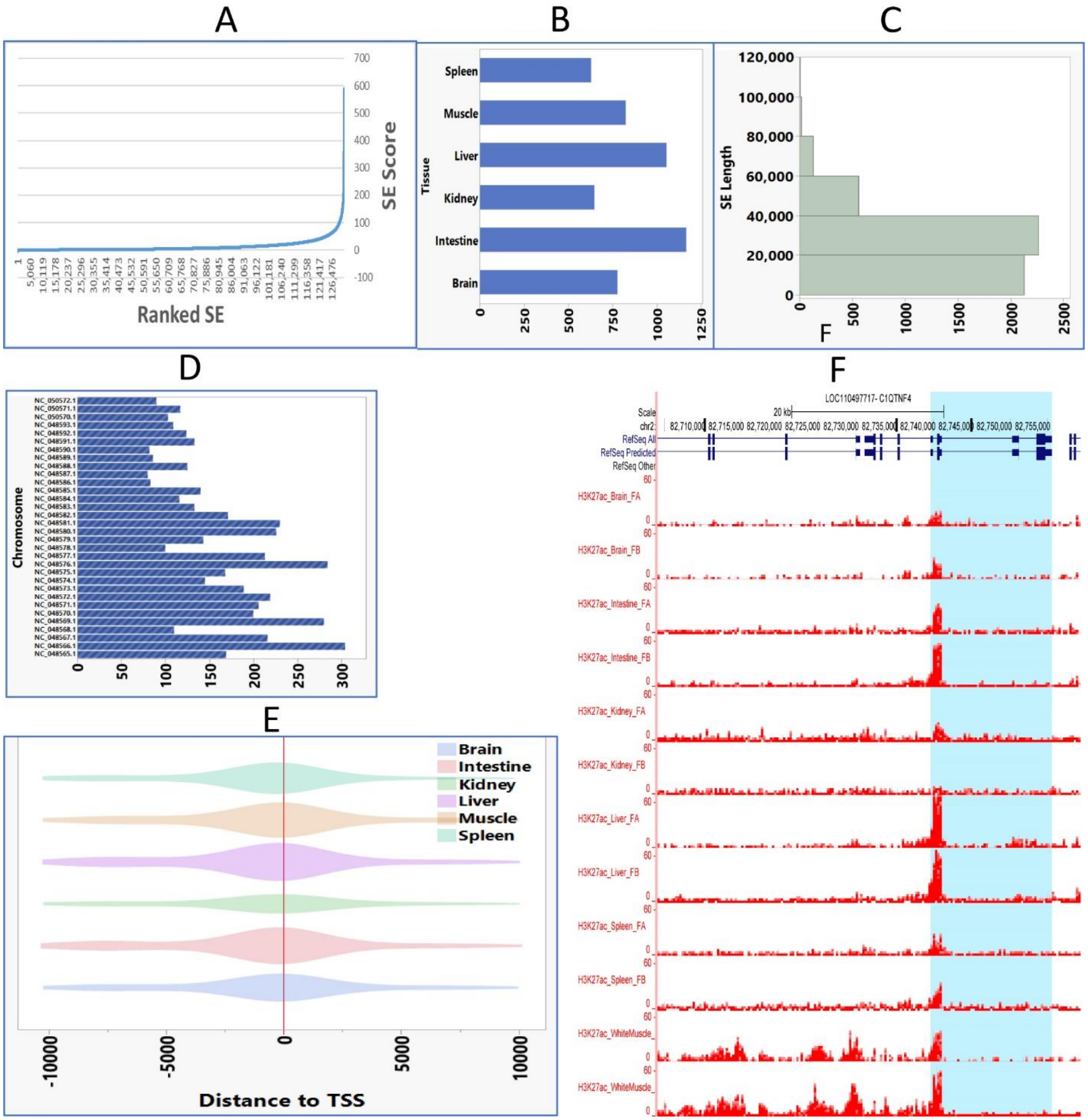

Figure 3. Super Enhancers and their characterization. (A) Ranked SE identified by HOMER based on an extremely high H3K27ac signal compared to conventional enhancers. **(B)** Number of SE in each tissue. **(C)** SE length distribution. **(D)** Chromosome distribution of the SE. **(E)** SE are enriched around the gene TSS. **(F)** Example of a super enhancer with H3K27ac signal flanking the C1QTNF4 gene in muscle compared to a typical enhancer in all other tissues.

ENHANCERS IN QTL

To demonstrate the utility of the new chromatin annotations in identifying potential causal variants for complex phenotypic traits important for domestication, we cross-matched previously identified QTL in the RBT genome with genome tracks of the new gene regulatory elements, including promoters and enhancers. We used previously identified QTL with known genomic locations for fish growth, muscle yield, fillet quality, and bacterial cold water disease (BCWD) [33-38]. We identified 2,074 Str.Enh, overlapped with QTL-harboring genes located on 15 chromosomes, with mean and median overlap lengths of 1,524 and 1,000 bp, respectively (Figure 4A, Additional file 5). We also found 847 MidEnh overlapped with QTL-harboring genes located on 15 chromosomes, with mean and median overlapping lengths of 1,084 and 800 bp, respectively. Additionally, 3,975 EnhG enhancers overlapped with QTL-containing genes on all chromosomes, with mean and median overlap lengths of 874 and 600 bp, respectively (Additional file 4). Figure 4A&B shows the QTL and enhancers' fold enrichment (observed/expected) per chromosome. There were 124 fish/muscle growth and 84 BCWD unique QTL overlapping with 239 unique SE (Additional file 5).

To further investigate the epigenetic function of the SNPs in QTL, we looked at SNPs within QTL that overlap with the genic, strong, and mid-enhancers and have transcription factor binding motifs (TFBM). A total of 112 SNPs that met these criteria were located within 4 TFBM spanning 85 genes involved in fish/muscle growth, fillet quality, and BCWD (Figure 4C and Additional file 5). Interestingly, most TFBM (99%) were classified into only two families. The first TFBM family was C/EBP (with three TF members, C/EBP alpha, beta, and delta), making up 69.4% of the TFBM. The second TFBM family comprises glucocorticoid receptor (GR) and GR beta, constituting 30.1% of TFBM (Figure 4B). These data suggest a significant role of C/EBP and GR transcription factors in regulating fish/muscle growth, fillet quality, and BCWD.

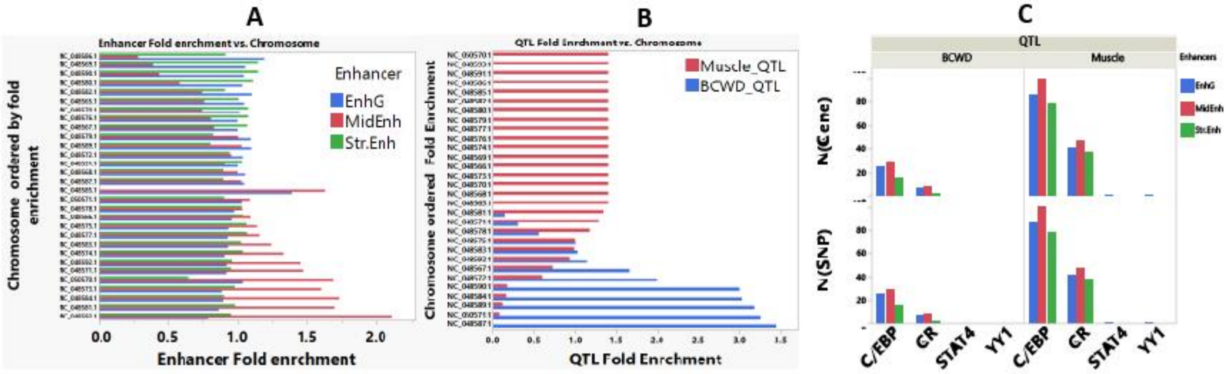

Figure 4. Enhancers in QTL. Fold enrichment per chromosome of active enhancers**(A)** that overlap with QTL**(B)** for fish/muscle growth, fillet quality, and BCWD. **(C)** Number of SNPs and genes within QTL that overlap with genic, strong, and mid-enhancers and have transcription factor binding motifs (TFBM) mainly belonging to glucocorticoid receptors (GR) and C/EBP transcription factors.

HISTONE MARK/STATE ROLE IN GENE EVOLUTION FOLLOWING WHOLE GENOME DUPLICATION

RBT is a member of the Salmonidae family that underwent a salmonid-specific whole genome duplication (Ss4R) 80-100 million years ago [39]. This WGD makes RBT an interesting model for studying the early stages of gene evolution. Therefore, we sought to identify the role of epigenomic chromatin marks and states in gene evolution following WGD and during the rediploidization of RBT.

We identified 20,660 gene duplicates inferred from collinear blocks in the RBT genome (See methods sections). We further identified 104 collinear blocks of at least 20 genes in the genome. Gene duplicates of RBT were then mapped against the Northern pike, which represents the ancestral singletons before duplication. We found 9,155 singletons in the Northern pike genome corresponding to 11,654 ohnologue pairs in RBT (Additional file 6). To distinguish the evolutionary processes that drive the preservation of gene duplicates after WGD, gene expression profile divergence was quantified among the duplicate pairs of RBT and ancestral genes of the Northern pike. The analysis revealed the presence of 73.6% gene conservation cases, 14.2% neofunctionalization cases, 12% specialization cases, and 0.2% subfunctionalization cases (Additional file 6).

We compared the fold enrichment of the histone marks and the abundance of chromatin states within the promoter region located 2Kb upstream of the TSS of each gene copy. Compared to neofunctionalized genes, there was less divergence in the histone modification profiles of conserved gene paralogues (Wilcoxon test, $P = 7.13E\text{-}270$) (Figure 5A). H3K27ac of the conserved gene pairs exhibited the highest correlation compared to H3K4me1 (Wilcoxon test, $P = 7.42E\text{-}99$) and H3K4me3 (Wilcoxon test, $P = 4.84E\text{-}08$). The H3K4me3 profile of the neofunctionalized gene pairs showed the most significant dissimilarity compared to the conserved genes (Wilcoxon test, $P = 1.07E\text{-}163$).

Similarly, the chromatin states in the promoter region of conserved gene pairs exhibited the highest correlation compared to neofunctionalized (Wilcoxon test, $P = 4.53E\text{-}46$) and specialized genes (Wilcoxon test, $P = 4.89E\text{-}11$) (Figure 5B). In addition, we observed less abundance of Str.Enh within the first seven chromatin states, upstream of the TSS of conserved genes. Except for BivEnh and RepPC, it was observed that the abundance of states upstream of the TSS was higher in gene pairs that are maintained through conservation (Wilcoxon test, $P < 2.2e\text{-}16$) (Figure 5C). Table 3 also shows the relative enrichment of all the chromatin states in each gene category. The single-copy genes had strong promoter and moderate signals, compared to the conserved genes which had strong promoter and enhancers' signals. The neofunctionalized and specialized genes had moderate enhancers' signals and very weak promoter signals.

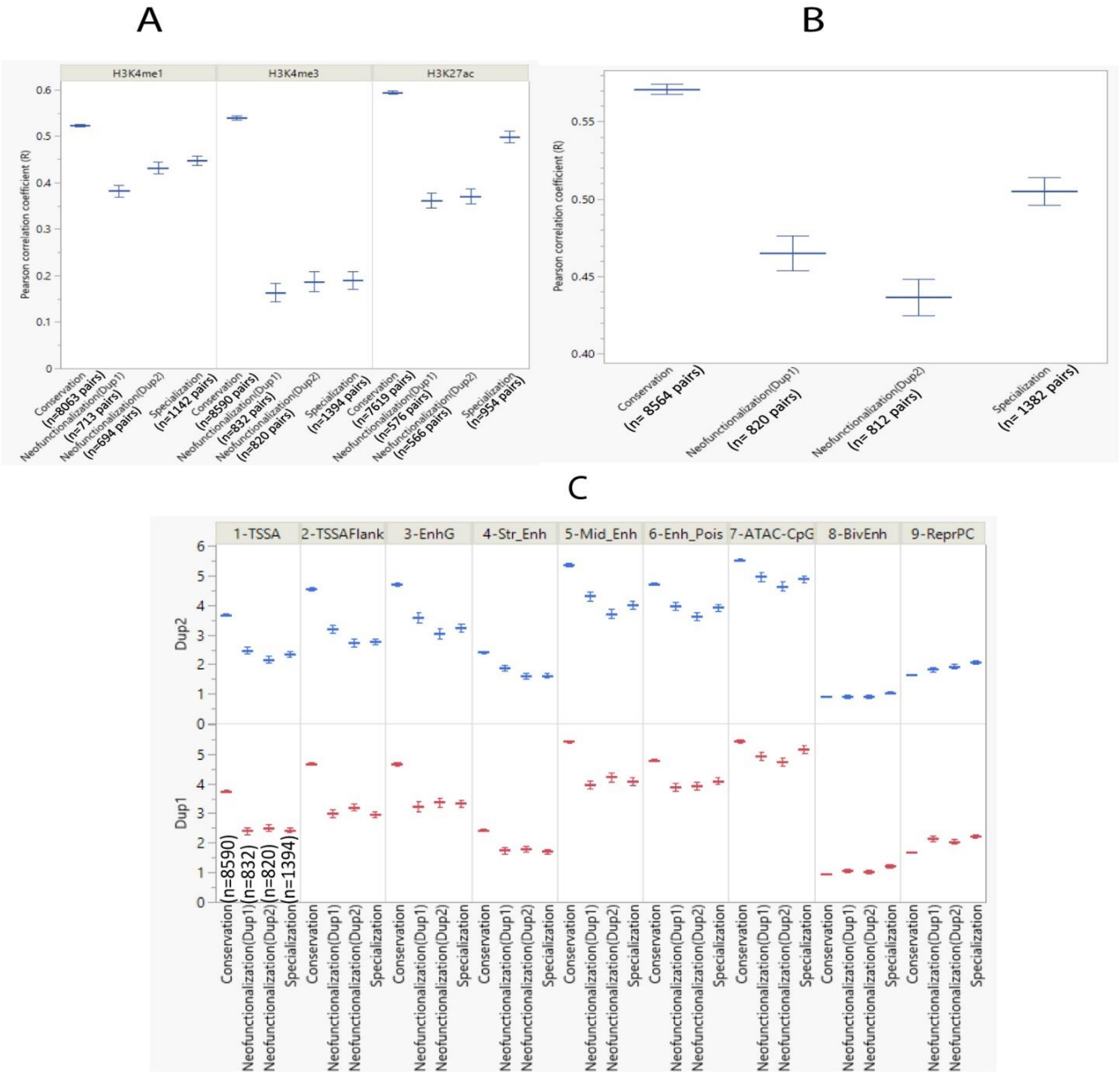

Figure 5. Association of the chromatin mark/state with the mechanism of duplicate gene retention. (A) The histone mark divergence of each category of the duplicate gene pair was quantified using the Pearson correlation coefficient of each histone mark profile for the duplicated gene pairs. Gene pairs with conserved expression exhibit the highest correlation of histone mark profiles upstream of TSS. **(B)** Correlation of state counts between gene pairs within each category. Gene pairs with conserved expressions demonstrate the highest correlation of state counts. **(C)** The shared number of states 1-7 within 2Kb upstream of the TSS is higher in conserved gene pairs. The shared numbers of Bivalent Enhancer (BivEnh) and Repressive Polycomb Complex (RepPC) showed a lower correlation.

Table 3. Relative enrichment of the chromatin states in the single-copy genes compared to the conserved genes, neofunctionalized and specialized genes

State	Single-copy	WGD	Conservation	Neofunctionalization	Specialization
TSSA	0.75	0.51	0.67	0.00	0.00
TSSAFlnk	0.82	0.41	0.52	0.09	0.08
EnhG	0.17	0.32	0.39	0.19	0.30
StrEnh	0.16	0.45	0.52	0.32	0.39
MidEnh	0.28	0.50	0.58	0.24	0.30
EnhPois	0.25	0.45	0.52	0.26	0.33
ATAC/CpG	0.14	0.05	0.12	0.00	0.06
BivEnh	0.08	0.03	0.07	0.01	0.13
ReprPC	0.00	0.00	0.00	0.09	0.16
Quies	0.16	0.12	0.19	0.09	0.10

DISCUSSION

The pioneering ENCODE projects built the foundations for discovering the regulatory element and their functions in humans and mammalian model species[20, 40, 41]. Following the ENCODE models, in the last decade, the Functional Annotation of Animal Genomes (FAANG) Consortium provided functional annotations atlas of the farm animal genomes, including pig, cattle, and chicken, for the first time [19, 27, 28]. However, functional annotations of fish genomes are still in their infancy, with comprehensive epigenomics tracks available perhaps only for zebra fish[29]. In the US, over the last ten years of the FAANG project, aquaculture was represented by one species, the RBT. As a part of the FAANG project, this study thus aimed to identify and characterize an atlas of regulatory elements and provide epigenome annotation tracks from RBT populations in the USA. We developed and characterized an atlas of regulatory elements and epigenome annotation tracks of the RBT. ChIP-seq, ATAC-seq, Methyl Mini-seq, and RNA-seq data were integrated across RBT tissues to identify gene regulatory elements, including chromatin histone modifications, chromatin accessibility, and DNA methylation.

This study identified regulatory elements, including 47,433 active promoters (19,784 TssA and 27,649 TssAFlnk). When this manuscript was ready for publication, the Ensemble genome browser released chromatin tracks for RBT, including promoters, enhances, and open chromatin

stats. For comparison, the Ensemble genome annotation browser has 23,394 promoters [42]. A total of 29,302 active promoters in our study were shared with promoters in the Ensemble genome browser (>100 nt). We also identified 80,404 active enhancers and 50,353 repressed enhancers, together (130,757) covering about 11.34% of the genome. The Ensemble genome annotation has 102,440 enhancers. Of all the enhancers identified in our study, 71,382 overlapped in genome positions with enhancers in the Ensemble genome browser (>100 nt). Variation in the numbers of the regulatory elements between our results and the Ensemble browser is expected due to differences in fish populations, tissues, physiological conditions, and the bioinformatics pipelines. In zebrafish, efforts to characterize the chromatin landscape identified 140,000 cis-regulatory elements [29]. And in mice, 33% of the genome had a chromatin signature of promoter, enhancer, transcriptional, and heterochromatin states[41].

In this study, the RBT active promoter and enhancer chromatin states were enriched around the genes TSS and TSS-flanking regions and zinc finger transcription factors and were highly transcribed but were depleted in the repressed genes (Figure 1). The RBT enhancers were also enriched in the expressed genes. Consistent with our results, the chicken genome promoters were more enriched in TSS, 5′UTR, and CpG islands than enhancers. The chicken active promoters and enhancers were more enriched in the TSS and the gene body of the highly expressed than the repressed genes[18].

This study also identified distinct patterns of DNA methylation associated with each chromatin state (Figure 1). All the active chromatin states (1-5) were hypomethylated compared to their flanking regions. On the other hand, the poised enhancers and quiescent genome regions were hypermethylated. The bivalent enhancers were strongly hypomethylated, the ATAC-CpG state was slightly hypermethylated, and the repressed polycomb showed no change in the methylation levels. Similar DNA methylation patterns were observed in the pig genome, where the promoter and the TSS transcribed states were hypomethylated, and the enhancer states showed intermediate methylation levels[19]. Previously, we also reported a sharp decline in DNA methylation within the ±2 kb of the TSS of the muscle genes[43].

We characterized the enrichment of the tissue-specific chromatin marks at promoter regions of tissue-specific expressed genes. H3K4me1 was enriched in the tissue-specific genes compared to the same genes in other tissues (silenced genes). On the other hand, H3K27me3 was enriched

in the tissue-silenced genes compared to the tissue-specific expressed genes (Figure 2). In addition, chromatin marks ATAC-Seq, H3K4me1, H3K4me3, and H27Kac were enriched in the expressed genes (>1 TPM), while H3K27me3 was enriched in promoters of the silenced genes (Figure 2). Similarly, the open chromatin states involving promoters and enhancers were more enriched in the genes with more than 1 TPM expression value. On the other hand, the repressed chromatin states RepPC, the ATAC-CpG, and BivEnh did not show characteristic density patterns relative to gene expression (Figure 2). Consistent with our results, in the pig genome, the active chromatin states (promoters, transcribed regions, and enhancers) were enriched in tissue-specific genes, while the repressed states were depleted [19]. In cattle, the relationships between chromatin states and gene expression showed that genes with TssA had the highest expression compared to genes with EnhPois, BivFlnk, and ReprPC [28].

Regarding the DNA methylation, we noticed a sharp decline in DNA methylation level within ±3 kb, flanking the genes' TSS. There was a trend of weak negative correlation between DNA methylation and gene expression, especially in the most highly expressed genes (long10 TPM > 3), and the correlation varies between tissues (Figure 2). These data confirm our previous reports showing a weak to moderate negative correlation between DNA methylation levels and gene transcription expression in muscle. The correlation was dependent on CpG position relative to TSS. The correlation was negative within ±1 kb of the TSS and positive in the gene body[43].

We have identified a total of 5,799 unique SEs in the RBT genome. Each tissue contained an average of 850.5 genes overlapping/neighboring SE (Figure 3B). The SE were generally shared between tissues, with only 805 (10.3%) SE exiting in a single tissue and the rest were shared between more than one tissue. 599 (13.8%) SE were ubiquitously existing in all tissues. SE in zebrafish showed more tissue specificity in four out of five tissues than regular enhancers[44].

Gene ontology analysis of the SEs' neighboring genes revealed functions relevant to essential molecular functions, including catalytic activity, DNA and metal/ion binding, and biological processes, including biosynthetic, cellular metabolic process, and transcription. SEs play a crucial role in determining cell identity and have been linked to the development of diseases [45] . Genes located within or near SEs had a higher gene expression than other genes, consistent with previous reports in mammals [46].

This study explored the potential epigenetic functions of previously identified QTL for complex phenotypic traits important for domestication by mapping QTL onto genome tracks of the regulatory elements. The active enhancer states (EnhG, Str.Enh, and MidEnh) and the EnhPois were enriched in genome regions spanning QTL. We identified 2,074 Str.Enh, 847 MidEnh, and 3,975 EnhG enhancers overlapped with QTL-containing genes on all chromosomes. Similar to our data, a recent study on cattle confirmed that active promoters/transcripts exhibited the highest enrichment for QTL. The cattle study also showed that weak enhancers had the highest enrichment for eQTLs compared to 14 other chromatin states[28].

We took a closer look to investigate the potential epigenetic functions of the SNPs in QTL that overlap with the enhancers and have transcription factor binding motifs (TFBM). Out of 108 SNP markers within 84 genes involved in fish/muscle growth, fillet quality, and BCWD, we identified 8 TFBM (Figure 4). Interestingly, almost all the TFBM (99%) were classified into only two families: the C/EBP and the glucocorticoid receptors (GR).

The glucocorticoid hormone is key in regulating muscle mass, and prolonged cell exposure to it causes muscle atrophy[47]. Muscle-specific deletion of GR in mice skeletal muscle increases muscle mass, reducing fat mass and muscle atrophy [48, 49]. Similarly, C/EBPβ is a central regulator of cancer muscle mass loss (cachexia) via promoting the expression of atrophy-inducing factors[50]. In RBT, stress increases cortisol levels and susceptibility to BCWD. A recent study found that rainbow trout BCWD-resistant fish are less sensitive to cortisol-induced IgM response than susceptible/control fish[51]. Another recent study by De Laval et al. revealed that short-term lipopolysaccharide-induced immune signaling can activate C/EBPβ-dependent chromatin accessibility, leading to trained immunity in hematopoietic stem cells during secondary infection. This establishes an epigenetic mechanism of memory function in innate immunity[52].

Our data regarding the C/EBP and GR warrant further studies to include CRISPR-Cas9 gene editing to confirm the causative nature of the SNPs involved in C/EBP and GR transcription factors and their role in regulating muscle growth, fillet quality, and BCWD. The muscle growth and quality and BCWD QTL analysis targeted in this study is an example of the potential utility of the genome annotation tracks generated as valuable tools in prioritizing genetic variants when searching for causal variants and alleles with major effects on domestication traits and genomic selection.

The ancestral genome of teleost fish underwent a teleost-specific third WGD (Ts3R), estimated to have occurred 225-333 million years ago[53], followed by the divergence of the Salmonidae family, which underwent a fourth salmonid-specific WGD (Ss4R), estimated to have occurred ~80-100 million years ago [39]. The recent salmonid-specific WGD and the existence of large genome segments as duplicate regions make RBT unique as a model organism to study the early stages of gene evolution. Therefore, we sought to identify the evolutionary processes that drive the preservation of gene duplicates and gain a better understanding of the role of epigenomes in gene evolution following WGD and during the rediploidization of RBT.

To distinguish the evolutionary processes that drive the preservation/neofunctionalization of gene duplicates after WGD, gene expression profile divergence was quantified among 11,654 ohnologue pairs in RBT and their ancestral singletons in the Northern pike. This phylogenetic approach was initially developed by Assis and Bachtrog [54]. The analysis revealed the presence of 73.6% gene conservation cases, 14.2% neo-functionalization cases, 12% specialization cases, and 0.2% subfunctionalization cases. These results indicate that conservation maintains the majority of the gene duplicates following WGD. In Atlantic salmon, Lien et al.[39] reported that 42% of the Ss4R duplicates displayed conserved co-expression with their orthologs in Northern Pike.

Enhancers and promoters predominantly enrich epigenetic signatures [55, 56]. We thus postulated that genes displaying noticeable variations in gene expression would also exhibit contrasting epigenetic patterns. To validate this hypothesis, we compared the fold enrichment of the histone marks and the abundance of chromatin states within the promoter region located 2Kb upstream of the TSS of each gene copy. Compared to neofunctionalized genes, there was less divergence in the histone modification profiles of conserved gene paralogues. H3K27ac of the conserved gene pairs exhibited the highest correlation compared to H3K4me1 and H3K4me3 (Figure 4A). In their recent study on Atlantic salmon, Verta et al. (2021) reported that the transcriptional divergence observed in duplicated genes resulting from WGD is found to be correlated with variations in the number of nearby regulatory elements, suggesting that the functional divergence between ohnologues following WGD is primarily driven by enhancers[57]. In this study, the H3K4me3 profile of the neofunctionalized gene pairs showed the most significant dissimilarity compared to the H3K4me3 profile of the conserved genes, which aligns with the

divergence observed in gene expression. Our results suggest a role for the promoters in the functional divergence between ohnologues following WGD.

Similarly, the chromatin states in the promoter region of conserved gene pairs exhibited the highest correlation compared to neofunctionalized and specialized genes, which may help explain their increased stability and conservation(Figure 5B). Furthermore, compared to other enhancers, we observed less abundance of Str.Enh upstream of the TSS in the conserved genes. Also, except for BivEnh and RepPC, the abundance of the chromatin state upstream of the TSS was higher in gene pairs maintained through conservation (Figure 5C). Together, our study reveals significant enrichment of distinct epigenetic signatures in ohnologue pairs exhibiting divergent gene expression modes.

Overall, this study provides a new atlas of regulatory elements in the RBT genome, which will help accelerate the genetic selection efforts, mainly through GWAS and genomic selections, to improve essential production traits in RBT for domestication. In addition, the new chromatin atlas will help in understanding the functional genomic basis of RBT's phenotypic, environmental, and evolutional variations.

METHODS

ANIMALS AND TISSUES

Six tissues (brain, intestine, liver, kidney, spleen, and white muscle) were collected at Washington State University, Dr. Gary Thorgaard's laboratory, from two individual doubled haploid Swanson clonal line fish. Tissues were flash-frozen in liquid nitrogen before being stored at –80 °C until further processing. The Institutional Animal Care and Use Committee at Washington State University reviewed and approved the animal study under protocol #02456.

CHIP-SEQ AND ATAC-SEQ

ChIP-seq (H3K4me3, H3K27ac, H3K4me1, and H3K27me3) library preparations were performed using the iDeal ChIP-seq kit (Diagenode Cat.#C01010059, Denville, NJ), as previously described[18, 19]. In brief, approximately 20–30 mg powdered tissue was cross-linked using 1% formaldehyde for 8 min before quenching with 100 µl of glycine for 10 min. Cell nuclei were isolated by centrifugation at 2000×g for 5 min, resuspended in 600 µl of iS1 buffer, and incubated on ice for 30 min. Chromatin was sheared using a Bioruptor Pico for 10 to 15 cycles, depending

on the tissues. For immunoprecipitation, about 1–1.5 µg of sheared chromatin was used as input with 1 µg of the specific histone mark antibody according to the manufacturer protocol: H3K4me3 (part of the Diagenode iDeal Histone kit #C01010059), H3K27me3 (#C15410069), H3K27ac (#C15410174), H3K4me1 (#C15410037). An input with no antibody was used as a negative control for each sample. NEBNext Ultra DNA library prep kit (#E7645L) from New England Biolabs (Ipswich, MA). was used for library construction. Libraries were sequenced using an Illumina HiSeq 4000 platform (RRID:SCR_016386) with a single-end read length of 50 bp. Additionally, ATAC-seq libraries were prepared using a modified Omni-ATAC57 protocol on cryopreserved nuclei [58]. The DNA sequencing was performed on Illumina's NextSeq platform, with a 40bp paired-end read length. Sequencing reads were trimmed with Trim Galore (RRID:SCR_011847) (v.0.6.5)[59] and aligned with bowtie2 (RRID:SCR_016368)[60] (v.2.5.4a) to the RBT genome (NCBI Accession GCA_013265735.3), and then duplicates were marked using Picard (RRID:SCR_006525) (v.2.18.7). MACS2 was used to call regions of signal enrichment ("peaks") [61]. The correlations between assays, tissues, and biological replicates were performed by deepTools (RRID:SCR_016366) [62]

CHROMATIN STATE ANNOTATION

ChromHMM69 (v.1.20) was used to predict the chromatin state by integrating ChIP-seq (H3K4me3, H3K4me1, H3K27ac, H3K27me3, and input control) from two biological replicates of all 6 tissues and ATAC-seq data from three tissues (brain, liver, and spleen). A 10-state model was chosen to represent the most appropriate number of distinct states based on the histone marks and accessibility combinations and their enrichment[18, 19]. In addition, the fold enrichment of each chromatin state for each gene annotation element (e.g., TSS, 5'UTR, and QTL) was calculated by (C/A)/(B/D), where A, B, C, D are the number of bases in a chromatin state, a gene element, overlapped between a chromatin state and a gene element, in the genome, respectively.

RNA SEQUENCING DATA

RNA sequence data for the six tissues used in this study were downloaded from our previously described NCBI BioProject PRJNA389609. Sequence read mapping to genome reference and assessment of TPM expression values per gene was performed using the CLC genomics workbench (Qiagen Inc., Redwood City, CA, USA).

METHYL-MINISEQ

Genome-wide bisulfite library preparation and sequencing were done using the Methyl-MiniSeq® Service at Zymo Research (Irvine, CA, USA) as previously described[43]. Briefly, DNA was extracted using Quick-DNA Plus Miniprep Kit. Five hundred nanograms of genomic DNA were digested with 60 units of TaqαI followed by 30 units of MspI (NEB) and then purified with Zymo Research DNA Clean & Concentrator™-5. According to Illumina's guidelines, DNA fragments were ligated to adapters containing 5′-methylcytosine instead of cytosine. The adaptor-ligated fragments of 150–250 bp and 250–350 bp were retrieved from a 2.5% NuSieve 1:1 agarose gel using Zymoclean™ Gel DNA Recovery Kit. The EZ DNA Methylation-Lightning™ Kit was used for the bisulfite treatment. PCR was performed, and then the products were purified using DNA Clean & Concentrator™-5 for sequencing on an Illumina HiSeq.

Raw FASTQ files were adapter- and quality-trimmed using TrimGalore 0.6.5[59]. Filled-in nucleotides were also trimmed using TrimGalore 0.6.5. Reads with a quality < 20 were removed. Bismark 0.22.3 was used to align the sequence reads to the RBT genome (NCBI Accession GCA_013265735.3)[63]. The methylated and unmethylated read totals for each CpG site were retrieved using the Bismark Methylation Extractor. CpG sites with less than ten read depths or more than 99.9th percentile of coverage in each sample were filtered out to account for PCR bias. The methylation level of the cytosines was calculated as the number of reads calling C divided by the total number of reads calling C and T, as previously described[43]. JMP Pro®, Version 15. SAS Institute Inc. (Cary, NC, USA) was used to generate figures and statistical measures of the association between DNA methylation percent and gene transcription expression levels.

HISTONE MARKS CORRELATION WITH GENE EXPRESSION

To assess the enrichment of the chromatin marks and states around the TSS of the tissue-specific expressed genes among tissues, we first determined the TPM value of each gene in each tissue. The expression level of each gene in a specific tissue was compared to its expression level in all remaining tissues. For a gene to be tissue-specific genes, the fold-change in the expression level of the gene had to be ≥ 10 fold than the sum of the TPM values in all other tissues, or the TPM value of the gene had to be ≥ 1, and the rest of the other tissues are zero. The same genes were considered silenced genes in the other tissues (showing no or almost no expression) for comparison. Second, we identified the chromatin mark or state that uniquely exists in the tissue-

specific genes within ±3 kb of TSS in each gene. JMP Pro®, Version 15. SAS Institute Inc. (Cary, NC, USA) was used to generate figures and statistical measures of the association between gene transcription expression levels and densities of the chromatin marks and states.

IDENTIFICATION OF SUPER-ENHANCERS

The HOMER algorithm findPeaks tool was utilized to identify peaks and calculate ChIP-seq tags from the H3K27ac ChIP-seq bam files. The parameter of finding histone-enriched regions (-style histone) was used. H3K27ac enriched signals were used to identify enhancers[32, 64]. Enhancers that were located within 12.5 kb of each other were clustered together. The enhancer clusters were then ranked based on H3K27ac signals using the HOMER super-enhancer tool. Enhancers with a tangent slope greater than 1 were considered super-enhancers, while enhancers with a tangent slope less than or equal to 1 were considered conventional enhancers. Nonredundant super-enhancers were determined by merging (at least an overlap of 50% of SE length) across all tissues. Genes overlapped with SE were annotated for gene ontology molecular functions and biological processes using DAVID[65].

ENHANCERS AND TRANSCRIPTION FACTOR BINDING SITES IN QTL

Previously identified QTL associated with fish growth, muscle growth, fillet quality, and bacterial cold-water disease were used as gene elements in the chromatin state analyses explained above [33-38]. Genes overlapped with enhancer states in QTL were identified. Then, we searched for SNPs within QTL that overlap with the genic, strong, and mid-enhancers and are located within transcription factor binding motifs. The transcription factor binding motifs were identified by PROMO[22] using version 8.3 of TRANSFAC software. SNPs within these motifs that may affect transcription factor binding were identified. The most common motifs associated with fish/muscle growth and fillet quality traits were presented.

HISTONE MARK/STATE ROLE IN GENE EVOLUTION FOLLOWING WHOLE GENOME DUPLICATION

Identification of Genes in Collinear Blocks

The RBT protein sequences and genomic positions were obtained from the NCBI database (Accession number "GCA_013265735.3"). For genes with multiple transcripts, the transcript with the longest coding sequence (CDS) was selected. To determine homology, protein-coding genes were compared against themselves using BLASTp, specifically the All-vs.-All local BLASTp

approach. The top five hits, excluding self-hits, with an E-value threshold of less than 10^{-5} for each protein sequence were recorded. This process allowed for identifying potential homologous proteins across the rainbow trout genome.

The MCScanX software package[66] was utilized to categorize genes into five distinct types based on their copy number and genomic distribution. These types include singletons, dispersed duplicates, tandem duplicates, proximal duplicates, and WGD/segmental duplicates. To execute the duplicate gene classifier, a core program of MCScanX, the BLASTp output, and the annotation file were used as input files.

The classification of gene duplication was determined as follows: initially, all genes were labeled as singletons and assigned ranks based on their order on chromosomes. Genes that exhibited BLASTp hits to other genes were then relabeled as dispersed duplicates. Gene pairs were classified as proximal duplicates if their difference in gene rank was less than 20 (configurable) or as tandem duplicates if the difference in gene rank was equal to 1. Finally, the MCScanX program was executed, and anchor genes within collinear blocks were relabeled as segmental/WGD duplicates.

In cases where a gene appeared in multiple hits, it was assigned to a unique class based on the following order of priority: WGD/segmental duplicates, tandem duplicates, proximal duplicates, and dispersed duplicates.

Divergence of Histone Modifications

We first calculated the log2-transformed fold enrichment ratio. Then, we converted these ratios into z scores using the formula $Z_X = (\chi - \mu)/\delta$ as in [67]. In this equation, χ represents the ratio value for a specific gene, μ denotes the mean ratio of all genes, and δ signifies the standard deviation of this ratio across all genes.

To assess the correlation and divergence of histone modification patterns between duplicate gene pairs, we utilized the Pearson correlation coefficient “r” of the histone modification profiles for the duplicated gene pair and dissimilarity index (1-r), respectively. By comparing the mean values of “r” or “1-r” in each gene category, we determined the significance using the Wilcoxon rank-sum test.

Quantification of Gene Expression

To quantify gene expression, we obtained the raw RNA-seq reads of RBT (Acc# SRP108798) and Northern pike (Acc# SRP040114) from the NCBI SRA database. To ensure data quality, these raw reads were then subjected to trimming using the CLC Genomics Workbench (version 22.0).

Next, we mapped the high-quality reads to the reference genome sequence (GCF_013265735.2) using the HISAT2 aligner[68]. To retrieve the abundance levels of each gene, we utilized the BAM files and employed the TPMCalculator to calculate the gene expression levels based on the number of uniquely mapped reads to each gene.

Identification of the Mechanisms of Duplicate Gene Preservation

The WGD duplicates, obtained from the output file that contains collinear blocks identified by MCScanX (RRID:SCR_022067) [66], were subjected to a blast analysis against non-collinear genes from the Northern pike. If both members of the duplicate gene-pair matched the same singleton (with an E-value < 10^{-5}), the gene triplet was selected for further downstream analysis.

We limited our analyses to triplets, where every gene copy is expressed in at least one tissue. To determine the expression prior to duplication, we used the singletons' expression profile in male Northern pike as a proxy. All absolute expression levels were then converted into relative expression levels, representing the proportions of contributions to total expression. These relative expression values were employed as gene expression profiles for comparison.

We employed the phylogenetic method developed by Assis and Bachtrog[54, 69] to categorize the evolutionary processes and mechanisms that retain pairs of duplicate genes. To determine the preservation of these duplicates, we calculated the Euclidean distances between the expression profiles of D1 and ancestral copies ($E_{D1,A}$), D2 and ancestral copies ($E_{D2,A}$), and the combined D1-D2 expression profile and that of the ancestral copy ($E_{D1+D2,A}$). To establish a baseline level of gene divergence, we also calculated the Euclidean distances between the expression profiles of singletons in sister species ($E_{S1,S2}$). We explored various cutoff values to define expression divergence and ultimately selected the semi-interquartile range from the median due to its robustness to outliers. Based on previously established rules, we classified each pair of duplicates as conserved, neofunctionalized, subfunctionalized, or specialized. In cases where

duplicates are conserved, we expect $E_{D1,A} \leq E_{S1,S2}$ and $E_{D2,A} \leq E_{S1,S2}$. For neofunctionalization of D1, we anticipate $E_{D1,A} > E_{S1,S2}$ and $E_{D2,A} \leq E_{S1,S2}$. Similarly, for neofunctionalization of D2, we expect $E_{D1,A} \leq E_{S1,S2}$ and $E_{D2,A} > E_{S1,S2}$. In cases where duplicates are subfunctionalized, we anticipate $E_{D1,A} > E_{S1,S2}$, $E_{D2,A} > E_{S1,S2}$, and $E_{D1+D2,A} \leq E_{S1,S2}$. Finally, for the specialized duplicates, we anticipate that $E_{D1,A}$, $E_{D2,A}$, and $E_{D1+D2,A}$ are all greater than $E_{S1,S2}$.

Proof reading

Grammarly (2024) was used for text improving and proof reading[70].

DATA AVAILABILITY

RNA sequence data for the six tissues used in this study are available via the NCBI BioProjects PRJNA389609. The ChIP-seq and ATAC-seq data have been submitted to the NCBI Geo database under accession numbers. GSE245212. The epigenome state and marks annotation tracks are available through the UCSC genome browser GCF_013265735.2. Additional files are openly available in the OSF Platform [71]. All additional supporting data are available in the *GigaScience* repository, GigaDB [72].

FUNDING

This study was supported by competitive grants No, 2020-67015-30770, 2021-67015-33388, 2023-67015-39742 from the United States Department of Agriculture, National Institute of Food and Agriculture (MS).

CONTRIBUTIONS

MS and HZ designed the research. AA, LA, YW, XB, and YB performed the experiments. RA analyzed the data; MS wrote the manuscript. MS and RA contributed equally to the research.

CORRESPONDING AUTHOR

Correspondence to mosalem@umd.edu.

ETHICS APPROVAL AND CONSENT TO PARTICIPATE

Fish tissues were collected at Washington State University, Dr. Gary Thorgaard's laboratory, from two individual doubled haploid Swanson clonal line fish. The Institutional Animal Care and Use Committee at Washington State University reviewed and approved the animal study under protocol #02456.

CONSENT FOR PUBLICATION

Not applicable.

COMPETING INTERESTS

The authors declare that they have no competing interests.

Additional Fille 1: Overview of the sequencing dataset, QC and enrichment of histone marks/ states in tissue-specific genes versus silenced genes.

Additional Fille 2: Density of each chromatin state relative to the position of TSS of the protein-coding genes and correlation between DNA methylation and gene expression.

Additional Fille 3: Association of histone marks within ±3kb of TSS to gene expression.

Additional Fille 4: Supper enhancers.

Additional Fille 5: Enhancers, super enhancers, TFBM in QTL

Additional Fille 6: Retention mechanisms for rainbow trout gene duplicates-73.6% gene conservation cases, 14.2% neofunctionalization cases, 12% specialization cases, and 0.2% subfunctionalization cases.

Additional file 7: Description file showing the location of each chromatin state by chromosome.

REFERENCES

1. Thorgaard GH, Bailey GS, Williams D, Buhler DR, Kaattari SL, Ristow SS, et al. Status and opportunities for genomics research with rainbow trout. Comp Biochem Physiol B Biochem Mol Biol. 2002;133 4:609-46. doi:10.1016/s1096-4959(02)00167-7.
2. DJ H. Aquaculture Outlook. In: Service EORftER, (ed.). 2006.
3. Aquaculture Genomics G, Breeding W, Abdelrahman H, ElHady M, Alcivar-Warren A, Allen S, et al. Aquaculture genomics, genetics and breeding in the United States: current status, challenges, and priorities for future research. BMC Genomics. 2017;18 1:191. doi:10.1186/s12864-017-3557-1.
4. Gao G, Magadan S, Waldbieser GC, Youngblood RC, Wheeler PA, Scheffler BE, et al. A long reads-based de-novo assembly of the genome of the Arlee homozygous line reveals

chromosomal rearrangements in rainbow trout. G3 (Bethesda). 2021;11 4 doi:10.1093/g3journal/jkab052.

5. Pearse DE, Barson NJ, Nome T, Gao G, Campbell MA, Abadia-Cardoso A, et al. Sex-dependent dominance maintains migration supergene in rainbow trout. Nat Ecol Evol. 2019;3 12:1731-42. doi:10.1038/s41559-019-1044-6.

6. Elgin SC. The formation and function of DNase I hypersensitive sites in the process of gene activation. J Biol Chem. 1988;263 36:19259-62.

7. Thurman RE, Rynes E, Humbert R, Vierstra J, Maurano MT, Haugen E, et al. The accessible chromatin landscape of the human genome. Nature. 2012;489 7414:75-82. doi:10.1038/nature11232.

8. Stergachis AB, Neph S, Sandstrom R, Haugen E, Reynolds AP, Zhang M, et al. Conservation of trans-acting circuitry during mammalian regulatory evolution. Nature. 2014;515 7527:365-70. doi:10.1038/nature13972.

9. Rendeiro AF, Schmidl C, Strefford JC, Walewska R, Davis Z, Farlik M, et al. Chromatin accessibility maps of chronic lymphocytic leukaemia identify subtype-specific epigenome signatures and transcription regulatory networks. Nat Commun. 2016;7:11938. doi:10.1038/ncomms11938.

10. Honda BM, Candido PM and Dixon GH. Histone methylation. Its occurrence in different cell types and relation to histone H4 metabolism in developing trout testis. J Biol Chem. 1975;250 22:8686-9.

11. Bernstein BE, Humphrey EL, Erlich RL, Schneider R, Bouman P, Liu JS, et al. Methylation of histone H3 Lys 4 in coding regions of active genes. Proc Natl Acad Sci U S A. 2002;99 13:8695-700. doi:10.1073/pnas.082249499.

12. Santos-Rosa H SR, Bannister AJ, Sherriff J, Bernstein BE, Emre NC, Schreiber SL, Mellor J, Kouzarides T. Active genes are tri-methylated at K4 of histone H3. Nature. 2002;419 6905:407-11.

13. Shen Y, Yue F, McCleary DF, Ye Z, Edsall L, Kuan S, et al. A map of the cis-regulatory sequences in the mouse genome. Nature. 2012;488 7409:116-20. doi:10.1038/nature11243.

14. Xiao S, Xie D, Cao X, Yu P, Xing X, Chen CC, et al. Comparative epigenomic annotation of regulatory DNA. Cell. 2012;149 6:1381-92. doi:10.1016/j.cell.2012.04.029.

15. Heintzman ND, Stuart RK, Hon G, Fu Y, Ching CW, Hawkins RD, et al. Distinct and predictive chromatin signatures of transcriptional promoters and enhancers in the human genome. Nat Genet. 2007;39 3:311-8. doi:10.1038/ng1966.

16. Consortium EP. An integrated encyclopedia of DNA elements in the human genome. Nature. 2012;489 7414:57-74. doi:10.1038/nature11247.

17. Greer EL and Shi Y. Histone methylation: a dynamic mark in health, disease and inheritance. Nat Rev Genet. 2012;13 5:343-57. doi:10.1038/nrg3173.

18. Pan Z, Wang Y, Wang M, Wang Y, Zhu X, Gu S, et al. An atlas of regulatory elements in chicken: A resource for chicken genetics and genomics. Sci Adv. 2023;9 18:eade1204. doi:10.1126/sciadv.ade1204.

19. Pan Z, Yao Y, Yin H, Cai Z, Wang Y, Bai L, et al. Pig genome functional annotation enhances the biological interpretation of complex traits and human disease. Nat Commun. 2021;12 1:5848. doi:10.1038/s41467-021-26153-7.

20. Consortium EP, Moore JE, Purcaro MJ, Pratt HE, Epstein CB, Shoresh N, et al. Expanded encyclopaedias of DNA elements in the human and mouse genomes. Nature. 2020;583 7818:699-710. doi:10.1038/s41586-020-2493-4.

21. Gerstein MB, Lu ZJ, Van Nostrand EL, Cheng C, Arshinoff BI, Liu T, et al. Integrative analysis of the Caenorhabditis elegans genome by the modENCODE project. Science. 2010;330 6012:1775-87. doi:10.1126/science.1196914.
22. Messeguer X, Escudero R, Farre D, Nunez O, Martinez J and Alba MM. PROMO: detection of known transcription regulatory elements using species-tailored searches. Bioinformatics. 2002;18 2:333-4. doi:10.1093/bioinformatics/18.2.333.
23. mod EC, Roy S, Ernst J, Kharchenko PV, Kheradpour P, Negre N, et al. Identification of functional elements and regulatory circuits by Drosophila modENCODE. Science. 2010;330 6012:1787-97. doi:10.1126/science.1198374.
24. Wan ZY, Xia JH, Lin G, Wang L, Lin VC and Yue GH. Genome-wide methylation analysis identified sexually dimorphic methylated regions in hybrid tilapia. Sci Rep. 2016;6:35903. doi:10.1038/srep35903.
25. Beemelmanns A, Ribas L, Anastasiadi D, Moraleda-Prados J, Zanuzzo FS, Rise ML, et al. DNA Methylation Dynamics in Atlantic Salmon (Salmo salar) Challenged With High Temperature and Moderate Hypoxia. Frontiers in Marine Science. 2021;7 doi:10.3389/fmars.2020.604878.
26. Anastasiadi D, Diaz N and Piferrer F. Small ocean temperature increases elicit stage-dependent changes in DNA methylation and gene expression in a fish, the European sea bass. Sci Rep. 2017;7 1:12401. doi:10.1038/s41598-017-10861-6.
27. Kern C, Wang Y, Xu X, Pan Z, Halstead M, Chanthavixay G, et al. Functional annotations of three domestic animal genomes provide vital resources for comparative and agricultural research. Nat Commun. 2021;12 1:1821. doi:10.1038/s41467-021-22100-8.
28. Fang L, Liu S, Liu M, Kang X, Lin S, Li B, et al. Functional annotation of the cattle genome through systematic discovery and characterization of chromatin states and butyrate-induced variations. BMC Biol. 2019;17 1:68. doi:10.1186/s12915-019-0687-8.
29. Baranasic D, Hortenhuber M, Balwierz PJ, Zehnder T, Mukarram AK, Nepal C, et al. Multiomic atlas with functional stratification and developmental dynamics of zebrafish cis-regulatory elements. Nat Genet. 2022;54 7:1037-50. doi:10.1038/s41588-022-01089-w.
30. Johnston IA, Kent MP, Boudinot P, Looseley M, Bargelloni L, Faggion S, et al. Advancing fish breeding in aquaculture through genome functional annotation. Aquaculture. 2024;583:740589. doi:10.1016/j.aquaculture.2024.740589.
31. van der Velde A, Fan K, Tsuji J, Moore JE, Purcaro MJ, Pratt HE, et al. Annotation of chromatin states in 66 complete mouse epigenomes during development. Commun Biol. 2021;4 1:239. doi:10.1038/s42003-021-01756-4.
32. Heinz S, Benner C, Spann N, Bertolino E, Lin YC, Laslo P, et al. Simple combinations of lineage-determining transcription factors prime cis-regulatory elements required for macrophage and B cell identities. Mol Cell. 2010;38 4:576-89. doi:10.1016/j.molcel.2010.05.004.
33. Ali A, Al-Tobasei R, Lourenco D, Leeds T, Kenney B and Salem M. Genome-Wide Association Study Identifies Genomic Loci Affecting Filet Firmness and Protein Content in Rainbow Trout. Frontiers in Genetics. 2019;10 386 doi:10.3389/fgene.2019.00386.
34. Ali A, Al-Tobasei R, Lourenco D, Leeds T, Kenney B and Salem M. Genome-wide identification of loci associated with growth in rainbow trout. BMC Genomics. 2020;21 1:209. doi:10.1186/s12864-020-6617-x.

35. Salem M, Al-Tobasei R, Ali A, Lourenco D, Gao G, Palti Y, et al. Genome-Wide Association Analysis With a 50K Transcribed Gene SNP-Chip Identifies QTL Affecting Muscle Yield in Rainbow Trout. Front Genet. 2018;9:387. doi:10.3389/fgene.2018.00387.
36. Ali A, Al-Tobasei R, Lourenco D, Leeds T, Kenney B and Salem M. Genome-wide scan for common variants associated with intramuscular fat and moisture content in rainbow trout. BMC Genomics. 2020;21 1:529. doi:10.1186/s12864-020-06932-0.
37. Liu S, Martin KE, Gao G, Long R, Evenhuis JP, Leeds TD, et al. Identification of Haplotypes Associated With Resistance to Bacterial Cold Water Disease in Rainbow Trout Using Whole-Genome Resequencing. Front Genet. 2022;13:936806. doi:10.3389/fgene.2022.936806.
38. Vallejo RL, Evenhuis JP, Cheng H, Fragomeni BO, Gao G, Liu S, et al. Genome-wide mapping of quantitative trait loci that can be used in marker-assisted selection for resistance to bacterial cold water disease in two commercial rainbow trout breeding populations. Aquaculture. 2022;560:738574. doi:https://doi.org/10.1016/j.aquaculture.2022.738574.
39. Lien S, Koop BF, Sandve SR, Miller JR, Kent MP, Nome T, et al. The Atlantic salmon genome provides insights into rediploidization. Nature. 2016;533 7602:200-5. doi:10.1038/nature17164.
40. Breschi A, Munoz-Aguirre M, Wucher V, Davis CA, Garrido-Martin D, Djebali S, et al. A limited set of transcriptional programs define major cell types. Genome Res. 2020;30 7:1047-59. doi:10.1101/gr.263186.120.
41. Gorkin DU, Barozzi I, Zhao Y, Zhang Y, Huang H, Lee AY, et al. An atlas of dynamic chromatin landscapes in mouse fetal development. Nature. 2020;583 7818:744-51. doi:10.1038/s41586-020-2093-3.
42. Harrison PW, Amode MR, Austine-Orimoloye O, Azov AG, Barba M, Barnes I, et al. Ensembl 2024. Nucleic Acids Res. 2024;52 D1:D891-D9. doi:10.1093/nar/gkad1049.
43. Salem M, Al-Tobasei R, Ali A and Kenney B. Integrated Analyses of DNA Methylation and Gene Expression of Rainbow Trout Muscle under Variable Ploidy and Muscle Atrophy Conditions. Genes (Basel). 2022;13 7 doi:10.3390/genes13071151.
44. Perez-Rico YA, Boeva V, Mallory AC, Bitetti A, Majello S, Barillot E, et al. Comparative analyses of super-enhancers reveal conserved elements in vertebrate genomes. Genome Res. 2017;27 2:259-68. doi:10.1101/gr.203679.115.
45. Hnisz D, Abraham BJ, Lee TI, Lau A, Saint-Andre V, Sigova AA, et al. Super-enhancers in the control of cell identity and disease. Cell. 2013;155 4:934-47. doi:10.1016/j.cell.2013.09.053.
46. van Groningen T, Koster J, Valentijn LJ, Zwijnenburg DA, Akogul N, Hasselt NE, et al. Neuroblastoma is composed of two super-enhancer-associated differentiation states. Nat Genet. 2017;49 8:1261-6. doi:10.1038/ng.3899.
47. Watson ML, Baehr LM, Reichardt HM, Tuckermann JP, Bodine SC and Furlow JD. A cell-autonomous role for the glucocorticoid receptor in skeletal muscle atrophy induced by systemic glucocorticoid exposure. Am J Physiol Endocrinol Metab. 2012;302 10:E1210-20. doi:10.1152/ajpendo.00512.2011.
48. Braun TP, Grossberg AJ, Krasnow SM, Levasseur PR, Szumowski M, Zhu XX, et al. Cancer- and endotoxin-induced cachexia require intact glucocorticoid signaling in skeletal muscle. FASEB J. 2013;27 9:3572-82. doi:10.1096/fj.13-230375.

49. Yamazaki H, Uehara M, Yoshikawa N, Kuribara-Souta A, Yamamoto M, Hirakawa Y, et al. The crucial role of muscle glucocorticoid signaling in accelerating obesity and glucose intolerance via hyperinsulinemia. JCI Insight. 2023;8 8 doi:10.1172/jci.insight.162382.
50. AlSudais H, Rajgara R, Saleh A and Wiper-Bergeron N. C/EBPbeta promotes the expression of atrophy-inducing factors by tumours and is a central regulator of cancer cachexia. J Cachexia Sarcopenia Muscle. 2022;13 1:743-57. doi:10.1002/jcsm.12909.
51. Quddos F and Zwollo P. A BCWD-Resistant line of rainbow trout is less sensitive to cortisol implant-induced changes in IgM response as compared to a susceptible (control) line. Dev Comp Immunol. 2021;116:103921. doi:10.1016/j.dci.2020.103921.
52. de Laval B, Maurizio J, Kandalla PK, Brisou G, Simonnet L, Huber C, et al. C/EBPbeta-Dependent Epigenetic Memory Induces Trained Immunity in Hematopoietic Stem Cells. Cell Stem Cell. 2023;30 1:112. doi:10.1016/j.stem.2022.12.005.
53. Berthelot C, Brunet F, Chalopin D, Juanchich A, Bernard M, Noël B, et al. The rainbow trout genome provides novel insights into evolution after whole-genome duplication in vertebrates. Nature Communications. 2014;5 1:3657. doi:10.1038/ncomms4657.
54. Assis R and Bachtrog D. Neofunctionalization of young duplicate genes in Drosophila. Proc Natl Acad Sci U S A. 2013;110 43:17409-14. doi:10.1073/pnas.1313759110.
55. Zentner GE, Tesar PJ and Scacheri PC. Epigenetic signatures distinguish multiple classes of enhancers with distinct cellular functions. Genome Res. 2011;21 8:1273-83. doi:10.1101/gr.122382.111.
56. Papait R, Cattaneo P, Kunderfranco P, Greco C, Carullo P, Guffanti A, et al. Genome-wide analysis of histone marks identifying an epigenetic signature of promoters and enhancers underlying cardiac hypertrophy. Proceedings of the National Academy of Sciences. 2013;110 50:20164-9. doi:doi:10.1073/pnas.1315155110.
57. Verta J-P, Barton HJ, Pritchard V and Primmer CR. Genetic Drift Dominates Genome-Wide Regulatory Evolution Following an Ancient Whole-Genome Duplication in Atlantic Salmon. Genome Biology and Evolution. 2021;13 5 doi:10.1093/gbe/evab059.
58. Halstead MM, Kern C, Saelao P, Chanthavixay G, Wang Y, Delany ME, et al. Systematic alteration of ATAC-seq for profiling open chromatin in cryopreserved nuclei preparations from livestock tissues. Sci Rep. 2020;10 1:5230. doi:10.1038/s41598-020-61678-9.
59. Krueger F. Trim Galore: a wrapper tool around Cutadapt and FastQC to consistently apply quality and adapter trimming to FastQ files, with some extra functionality for MspI-digested RRBS-type (Reduced Representation Bisufite-Seq) libraries. UK2012. https://www.bioinformatics.babraham.ac.uk/projects/trim_galore/.
60. Langmead B and Salzberg SL. Fast gapped-read alignment with Bowtie 2. Nat Methods. 2012;9 4:357-9. doi:10.1038/nmeth.1923.
61. Zhang Y, Liu T, Meyer CA, Eeckhoute J, Johnson DS, Bernstein BE, et al. Model-based analysis of ChIP-Seq (MACS). Genome Biol. 2008;9 9:R137. doi:10.1186/gb-2008-9-9-r137.
62. Ramirez F, Ryan DP, Gruning B, Bhardwaj V, Kilpert F, Richter AS, et al. deepTools2: a next generation web server for deep-sequencing data analysis. Nucleic Acids Res. 2016;44 W1:W160-5. doi:10.1093/nar/gkw257.
63. Krueger F and Andrews SR. Bismark: a flexible aligner and methylation caller for Bisulfite-Seq applications. Bioinformatics. 2011;27 11:1571-2. doi:10.1093/bioinformatics/btr167.

64. Li X, Duan Y and Hao Y. Identification of super enhancer-associated key genes for prognosis of germinal center B-cell type diffuse large B-cell lymphoma by integrated analysis. BMC Med Genomics. 2021;14 1:69. doi:10.1186/s12920-021-00916-z.
65. Sherman BT, Hao M, Qiu J, Jiao X, Baseler MW, Lane HC, et al. DAVID: a web server for functional enrichment analysis and functional annotation of gene lists (2021 update). Nucleic Acids Res. 2022;50 W1:W216-W21. doi:10.1093/nar/gkac194.
66. Wang Y, Tang H, Debarry JD, Tan X, Li J, Wang X, et al. MCScanX: a toolkit for detection and evolutionary analysis of gene synteny and collinearity. Nucleic Acids Res. 2012;40 7:e49. doi:10.1093/nar/gkr1293.
67. Wang J, Orlov YL, Li X, Zhou Y, Liu Y, Yuan C, et al. In situ dissecting the evolution of gene duplication with different histone modification patterns based on high-throughput data analysis in Arabidopsis thaliana. PeerJ. 2021;9:e10426. doi:10.7717/peerj.10426.
68. Kim D, Paggi JM, Park C, Bennett C and Salzberg SL. Graph-based genome alignment and genotyping with HISAT2 and HISAT-genotype. Nature Biotechnology. 2019;37 8:907-15. doi:10.1038/s41587-019-0201-4.
69. Perry BR and Assis R. CDROM: Classification of Duplicate gene RetentiOn Mechanisms. BMC Evol Biol. 2016;16:82. doi:10.1186/s12862-016-0644-x.
70. Grammarly: https://app.grammarly.com/ (2024). 2024.
71. Salem M, Al-Tobasei R, Ali A, An L, Wang Y, Bai X, et al. Additional Files: Functional annotation of regulatory elements in rainbow trout uncovers roles of the epigenome in genetic selection and genome evolution. OSF. 2024; doi:10.17605/OSF.IO/87GYK.
72. Salem M, Al-Tobasei R, Ali A, An L, Wang Y, Bai X, et al. Supporting data for "Functional annotation of regulatory elements in rainbow trout uncovers roles of the epigenome in genetic selection and genome evolution" GigaScience Database. 2024. https://doi.org/10.5524/102592.

Figure 1

Click here to access/download;Figure;Figure 1 New.jpg

A

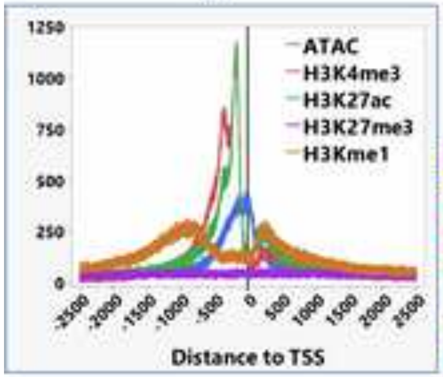

B C D E F

Chromatin State	Abbreviation	Emission					Genomic coverage	Fold enrichment of genomics annotations												
		ATAC	H3K4me3	H3K27ac	H3K4me1	H3K27me3		TSS	TSS-2Kb	5'UTR	Gene	Exon	TES	3'UTR	Expressed Genes	Repressed Genes	ZNF Genes	CpG Islands	QTL_ Muscle	QTL_ BCWD
1-Active TSS	TssA	0.79	0.98	0.95	0.22	0	0.79	0.98	0.94	0.99	0.52	0.85	0	0	0.79	0.06	0.53	0.14	0.61	0.39
2-Flanking Active TSS	TssAFlnk	0.33	0.87	0.13	0.37	0.02	0.63	0.85	0.85	0.6	0.44	0.59	0.14	0.06	0.6	0.14	0.76	0.16	0.48	0.34
3-Genic Enhancers	EnhG	0.96	0.01	0.14	0.16	0	1.2	0.24	0.21	0.1	0.29	0.09	0.53	0.44	0.37	0.05	0.11	0.21	0.53	0.38
4-Strong Enhancers	Str Enh	0.5	0.03	0.86	0.68	0	1.04	0.13	0.17	0.09	0.38	0.11	0.46	0.57	0.51	0	0.15	0	0.83	0.62
5-Mid Enhancers	MedEnh	0.1	0	0.07	0.69	0.01	1.62	0.1	0.27	0.08	0.43	0.15	0.5	0.55	0.53	0.01	0.3	0	0.77	0.67
6-Poised Enhancers	EnhPois	0.04	0	0.01	0.01	0	7.23	0.01	0.09	0.01	0.39	0.12	0.62	0.83	0.46	0.01	0.18	0.11	0.75	0.65
7-ATAC/CpG Island	ATAC/CpG	0.88	0.01	0	0	0.02	7.05	0.15	0.11	0.06	0.08	0	0.25	0.12	0.08	0.27	0.01	0.87	0.17	0.2
8-Bivalen Enhancers	BivEnh	0.94	0.29	0.18	0.24	0.71	0.25	0.12	0.14	0.12	0.06	0.08	0.19	0.01	0.04	0.5	0.02	0.92	0.04	0.1
9-Repressed PolyComb	ReprPC	0.05	0.01	0	0.01	0.42	2.57	0.09	0.2	0.08	0	0.08	0.45	0.29	0	0.68	0.01	0.84	0	0
10-Quiescent	Quies	0.01	0	0	0	0.01	77.64	0	0	0	0.13	0.01	0.23	0.35	0.1	0.32	0	0.22	0.27	0.33

G

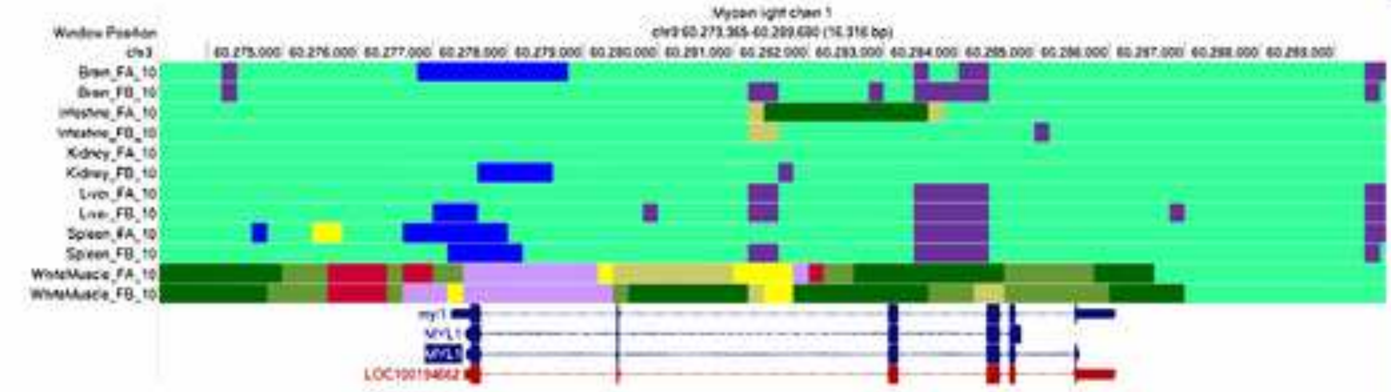

H

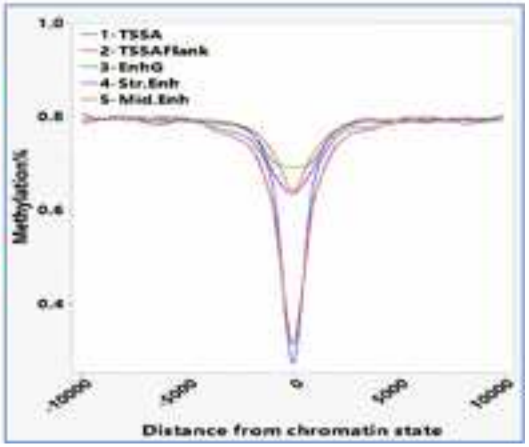

Figure 2

Click here to access/download;Figure;Figure 2 New.jpg

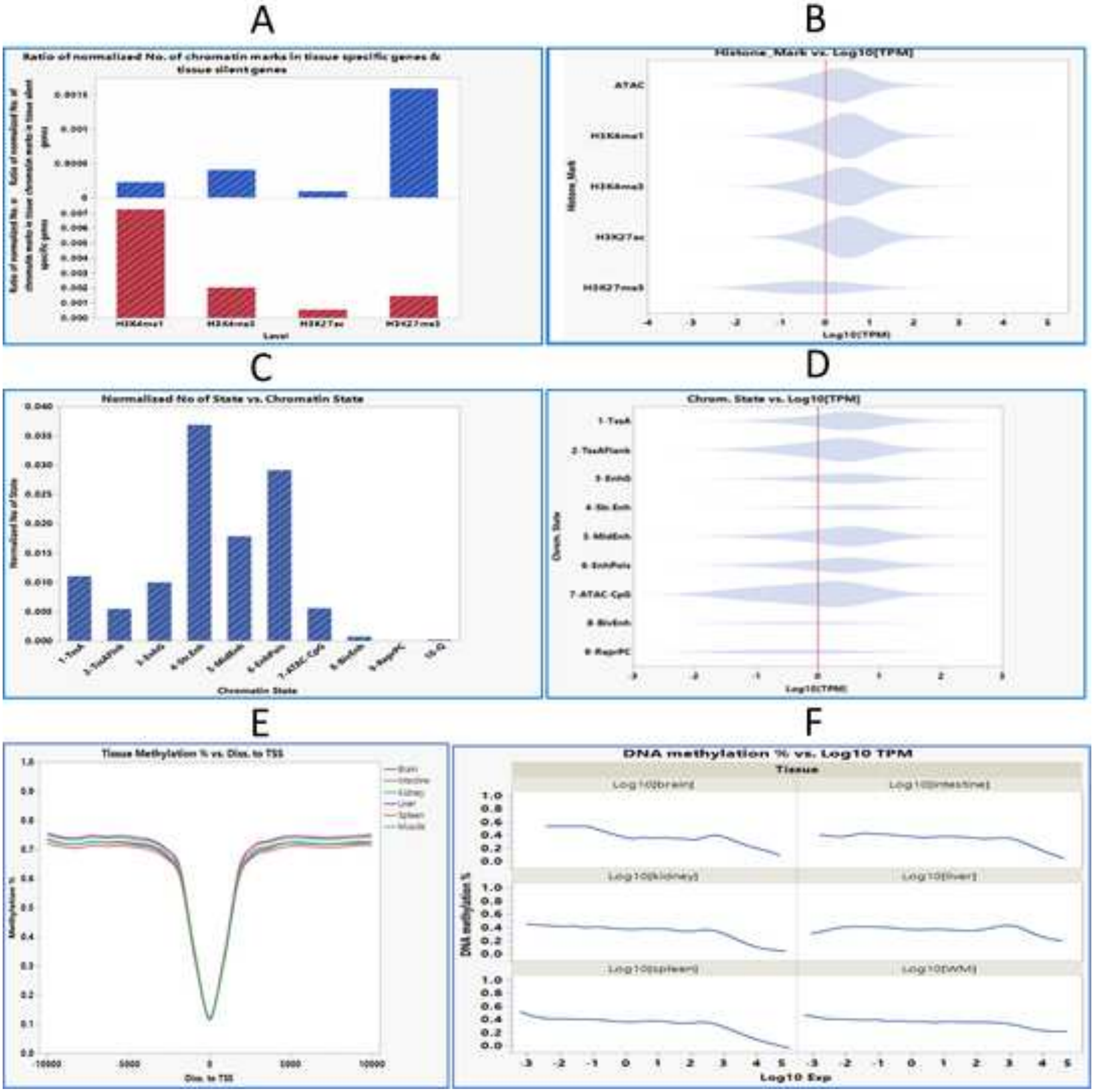

Figure 3

Click here to access/download;Figure;Figure 3 New.jpg

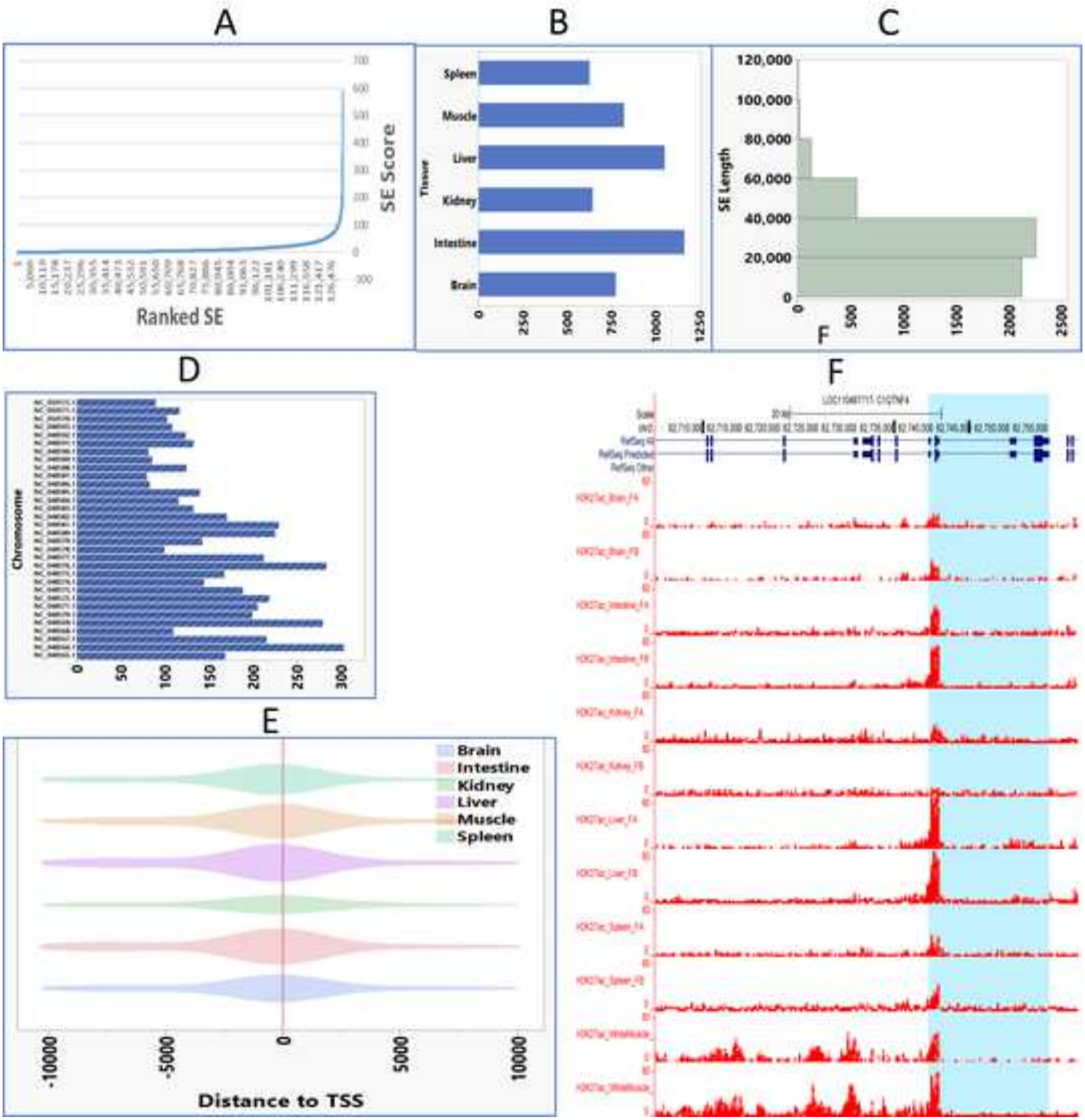

Figure 4

Click here to access/download;Figure;Figure 4 New2.jpg

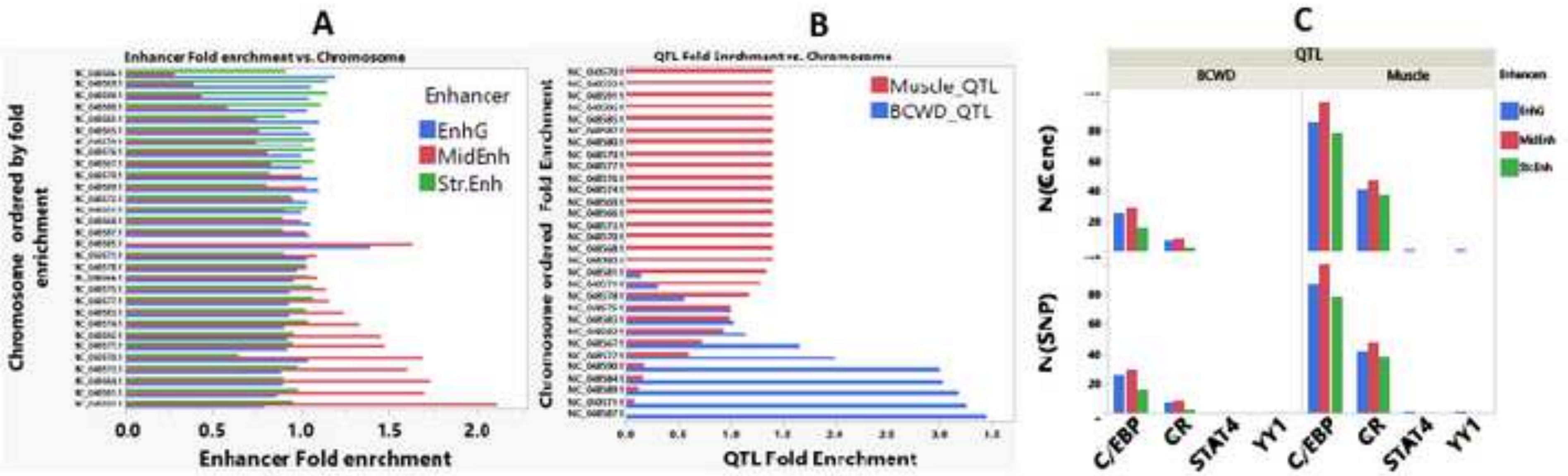

Figure 5

Click here to access/download;Figure;Figure 5 new.jpg

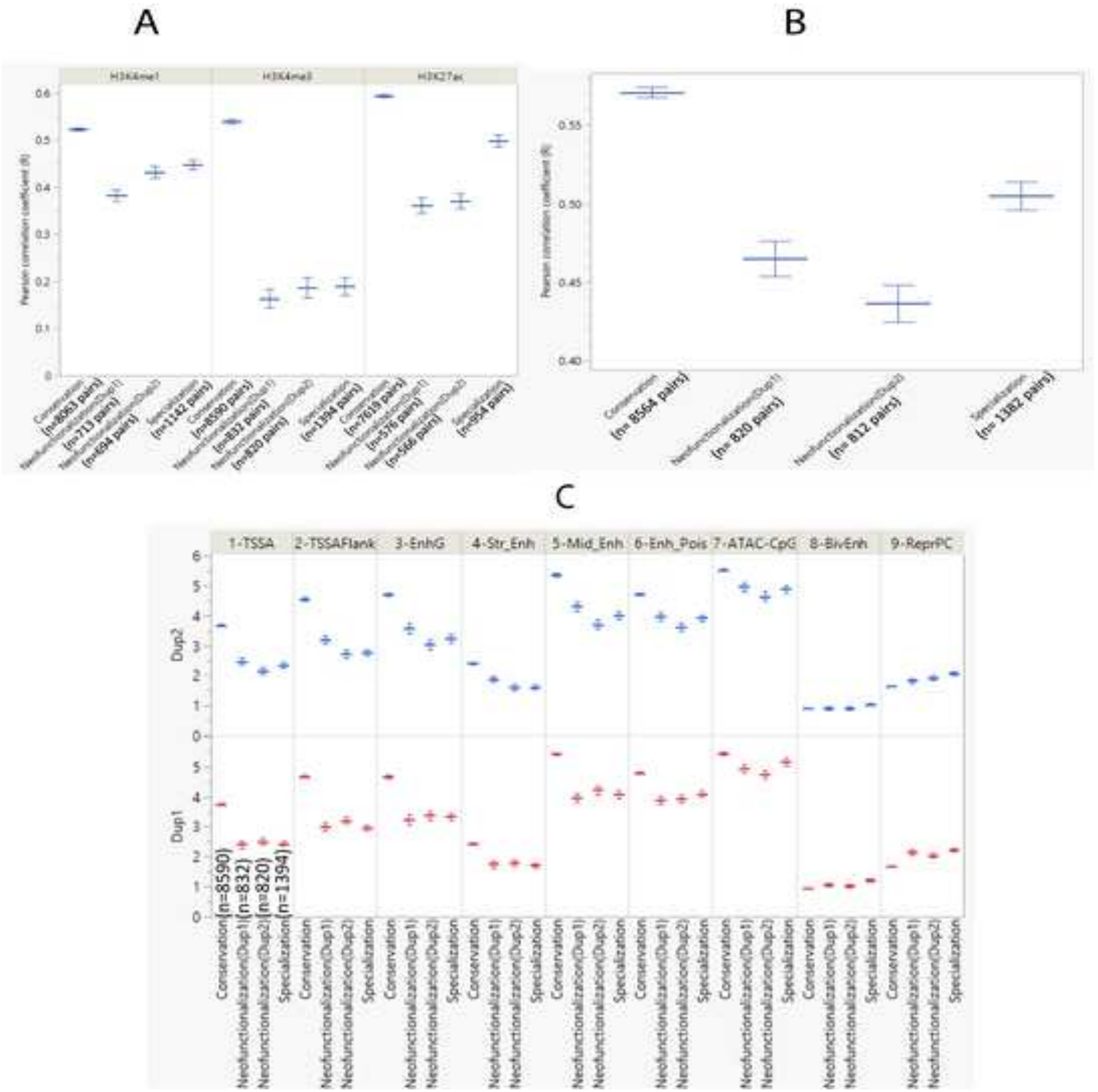

Additional File 1

Click here to access/download
Supplementary Material
Additional File_1.xlsx

Additional file 2

Click here to access/download
Supplementary Material
Additional File 2.pdf

Additional File 3

Click here to access/download
Supplementary Material
Addiotnal File 3.txt

Click here to access/download
Supplementary Material
Additional File 4.xlsx

Additional file 5

Click here to access/download
Supplementary Material
Additional File 5.xlsx

Additional file 6

Click here to access/download
Supplementary Material
Additional File 6.xlsx

Additional File 7

Click here to access/download
Supplementary Material
Additional File 7.txt

Response to reviewer comments
Click here to access/download;Personal Cover;Reviewer reports-Response2.pdf

Reviewer reports:

Reviewer #1: The manuscript from Salem et al. was greatly improved by the authors' providing the data and the code associated with processing and data analysis. The added QC shows that the data are good quality and suitable for subsequent analyses.

Response:

Dear Dr. Baranašić thank you for taking the time to review and improve the MS.

However, I still have some slight concerns about the ChromHMM annotations. The authors built their model based on three chromatin marks for all tissues and ATAC-seq signals from only three tissues. This approach resulted in a 10-state model with various emission probabilities. It is safe to assume that ChromHMM couldn't predict states with high ATAC emission in tissues where this assay is absent. However, looking at the ChromHMM annotation (Figure 1G), there is a strong enrichment in genic enhancers, characterised mainly by a strong ATAC signal and very little H3K27ac and H3K4me1. By inspecting the tracks provided in the genome browser (Please look at the Figure attached signal track browser screenshot and compare with Figure 1G annotation), I haven't found enough evidence that these regions would be enriched in the ATAC signal in white muscle tissue since there is no ATAC signal. The annotation probably stems from transition probabilities with other active states that could be observed.

My recommendation to the authors is to consider either building two separate ChromHMM models, one for tissues with ATAC signal and one for tissues without ATAC signal, or using imputation methods, such as ChromImpute, to estimate the presence of ATAC signal in the tissues missing it. In the latter case, the authors must demonstrate the effectiveness of the imputation. Implementing either of these approaches would help to avoid misleading annotations, such as the current one.

Response:

Dear Dr. Baranašić as your indicated, the ChromHMM tool is reliable for predicting states based on combinations of available chromatin tracks. In a study using the mouse ENCODE project data, ChromHMM was found to accurately assign chromatin states even when certain marks, including ATAC-seq, were missing. The research concluded that the absence of certain marks did not significantly impact the assignment. Although the states most affected were those most enriched in the missing mark in the ten-mark model, the opposite was not necessarily true, highlighting redundancy between the marks.

https://www.nature.com/articles/s42003-021-01756-4#Sec16

It is also important to recognize the complexity of checking chromatin states solely based on the original chromatin mark enrichment tracks. As the reviewer knows, determining chromatin states involves intricate mathematical calculations considering diverse combinations of chromatin marks and their corresponding negative controls. It's worth noting that among the FAANG consortium, not sure about Zebra fish and other model papers, our paper may stand out as the sole provider of UCSC chromatin state tracks for comparative analysis. In contrast, while extremely valuable,

other papers published in Nature and Science focused on providing only chromatin mark tracks without including chromatin state tracks.

https://www.nature.com/articles/s41467-021-26153-7

https://www.science.org/doi/10.1126/sciadv.ade1204

As a colleague note, during the review process of another manuscript from the FAANG consortium with a similar missing data, reviewers suggested combining data for chromatin state assignment, regardless of missing data. So, we think this is a subjective matter.

Any ways, to address this, we created a new 10-state chromatin model without ATAC-Seq data, and uploaded the bed files to the journal portal (Folder name: TroutChromHMM_State_without_ATAC_seq). We included an additional paragraph (below) in the manuscript to make the model available if the reader is interested.

We did a quick comparison of the enhancers count in case of using or without ATAC-seq data which revealed a 25% reduction in Genic Enhancers and a 23% increase in strong enhancers(See table below). Similarly, differences in the number of enhancers were reported in the mouse ENCODE paper.

	No. of Chromatin states			
	Genic Enh With ATAC-Seq	Genic Enh Without ATAC-Seq	Strong Enh With ATAC-Seq	Strong Enh Without ATAC-Seq
Muscle a	41,350	39,654	16,752	21,326
Muscle b	43,516	37,802	26,748	28,478
Spleen a	25,058	17,552	9,524	13,886
Spleen b	40,793	17,520	10,184	14,140
**Average**	**37,679**	**28,132**	**15,802**	**19,458**
% of change		-0.253382167		0.231331477

In addition, when looking at the MLC1 gene that you referred to in Figure 1G, some genic enhancers were still predicted, with even more other types of enhancers predicted in the absence of ATAC-Seq data.

	Chromatin states			
	With ATAC-Seq		Without ATAC-Seq	
Enhancer	Muscle Replicate A	Muscle Replicate A	Muscle Replicate A	Muscle Replicate A
EnhG	5	5	3	1
Str.Enh	3	1	3	2
MidEnh	1	0	2	0

EnhPois/WE	2	3	3	5
**Total**	**11**	**9**	**11**	**8**
Enhancer	Kidney Replicate A	Kidney Replicate A	Kidney Replicate A	Kidney Replicate A
EnhG	0	0	1	0
Str.Enh	0	0	0	0
MidEnh	0	0	0	0
EnhPois/WE	0	0	0	0
**Total**	**0**	**0**	**1**	**0**

We included this paragraph to address this issue in the MS.

The ATAC-seq data was missing in three tissues. The ChromHMM tool was found to be reliable for predicting states based on combinations of available chromatin tracks. In a study using the mouse ENCODE project data, ChromHMM accurately assigned chromatin states even when certain marks, such as ATAC-seq, were missing [31]. The research concluded that the absence of certain marks did not significantly impact the assignment. Although the states most affected were those most enriched in the missing mark in a ten-mark model, the opposite was not necessarily true, highlighting redundancy between the marks.

To address the effect of the missing ATAC-seq data in three tissues, we created a separate 10-state chromatin model without ATAC-seq data and uploaded the bed files to the journal portal (Folder name: TroutChromHMm_State_without_ATAC_seq. *A quick comparison of enhancers with and without ATAC-seq data revealed a 25% reduction in genetic enhancers and a 23% increase in strong enhancers using the muscle and kidney data. The mouse ENCODE paper reported similar differences in the number of enhancers [31]*

Finally, I suggest reorganising the track hubs. Using composite tracks for easier navigation and settings would greatly enhance the data's usability.

Response:

The tracks were reorganized as suggested, thank you.
