# Supplementary material for: Functional annotation of regulatory elements in rainbow trout uncovers roles of the epigenome in genetic selection and genome evolution
Source: Gigascience. 2024 Dec 4;13:giae092. doi: 10.1093/gigascience/giae092 (PMC11629980; doi:10.1093/gigascience/giae092)
Supplement: giae092_Response_to_Reviewer_Comments_Original_Submission [file giae092_response_to_reviewer_comments_original_submission.pdf]

Author's Response To Reviewer Comments

We alos included the Response to reviewers comments as attachment.

GIGA-D-24-00104
Functional annotation of regulatory elements in rainbow trout uncovers roles of the epigenome in genetic selection and genom[illegible]
Mohamed Salem; Rafet Al-Tobasei; Ali Ali; Liqi An; Ying Wang; Xuechen Bai; Ye Bi; Huaijun Zhou
GigaScience

Dear Professor Salem,

Your manuscript "Functional annotation of regulatory elements in rainbow trout uncovers roles of the epigenome in genetic se[illegible] genome evolution" (GIGA-D-24-00104) has been assessed by our reviewers. Although it is of interest, we are unable to cons[illegible] publication in its current form. The reviewers have raised a number of points which we believe would improve the manuscript[illegible] a revised version to be published in GigaScience.

Their reports, together with any other comments, are below. Please also take a moment to check our website at https://www.editorialmanager.com/giga/ for any additional comments that were saved as attachments.

If you are able to fully address these points, we would encourage you to submit a revised manuscript to GigaScience. Once y[illegible] the necessary corrections, please submit online at:

https://www.editorialmanager.com/giga/

If you have forgotten your username or password please use the "Send Login Details" link to get your login information. For s[illegible] your password will be reset.

Please include a point-by-point within the '--Response to Reviewers' box in the submission system. Please ensure you describ[illegible] experiments that were carried out and include a detailed rebuttal of any criticisms or requested revisions that you disagreed [illegible] ensure that your revised manuscript conforms to the journal style, which can be found in the Instructions for Authors on the [illegible] homepage. If the data and code has been modified in the revision process please be sure to update the public versions of thi[illegible]

In addition, we feel that you use Chat-GPT to write the manuscript as some parts of the Introduction and Discussion resembl[illegible] typical phrasing. Use of generative AI-writing tools such as ChatGPT is allowed as long as it is disclosed in a transparent man[illegible] not negatively affect empirical data and conclusions. AI-writing tools must be highlighted wherever necessary with in-text cita[illegible] mentioned among references. A summary of use (particularly in the introduction or among methods) needs to be included at [illegible] paper, and the outputs should also be included as a supplementary file hosted in GigaDB or other open repositories.

The due date for submitting the revised version of your article is 16 Sep 2024.

I look forward to receiving your revised manuscript soon.

Best wishes,

Hongfang Zhang
GigaScience
www.gigasciencejournal.com

--Response:
Dear Dr. Zhan,
Thank you for considering our manuscript for publication. We addressed all the reviewers' revisions and followed the instructi[illegible] use Chat-GPT in writing, we used Grammarly for proof reading and we added citations toward the end of the Methods section[illegible]

Reviewer reports:

Reviewer #1:
The manuscript titled "Functional annotation of regulatory elements in rainbow trout uncovers roles of the epigenome in gene
genome evolution" from Salem et al. provides an atlas of regulatory elements in six tissues of aquaculturally important rainbo
Although the authors provided a notable amount of novel data, I must raise major concerns regarding the data processing an
data analysis. My impression is that the analyses were often done without proper quality control or deeper consideration and
consultation, and most of the analyses should be rerun with some additional criteria in mind. I will address most of the issues
like to emphasise three main problems which recur throughout the manuscript:

--Response:
Dear Dr. Baranašić
We really appreciate taking the time to review and improve the MS, we have addressed all your revisions below and amended
accordingly.

a. The quality control of the raw and processed data is missing. It is hard to assess the quality of the analyses and annotation
considering how good the raw data are. The quality control should include at least a fraction of reads in peaks and some PCA
the data separate by tissue and reproducibility. Also, the authors should provide statistics on how well the peaks overlap betw
In addition, the peaks and the signal tracks must be available in the genome browser, along with all the annotations produce
manuscript.

----Response:
The raw data statistics were updated and provided as an additional file 1. Based on your suggestions, we added PCA/heatmap
description. The data/patterns are similar to previous data published in Nature and Science by Dr. Zhou's lab, which produced
and ATAC-Seq data https://www.nature.com/articles/s41467-021-26153-7
We apologize that the link to the signal tracks genome browser did not work for you. We made sure the link is working, and y
the tracks at
https://genome.ucsc.edu/cgi-
bin/hgTracks?hgS_doOtherUser=submit&hgS_otherUserName=Rafet&hgS_otherUserSessionName=GCF_013265735.2%2FG

b. The code for the analyses is not available. The code to reproduce the analyses in the manuscript should be publicly availab
some other public code repository.

--Response:
The code used in the analysis is now available at GitHub
https://github.com/rafet2005/Functional-annotation-of-regulatory-elements-in-rainbow-trout

c. It's crucial to emphasise that the Figure quality is currently low. Some screenshots have low-resolution labels that are hard
authors need to provide the figures as vector graphics of sufficient quality, as this will significantly enhance the clarity and im
research. Additionally, since the manuscript includes many profile plots and meta plots, adding a significance interval of the s
helpful to ensure that a small number of outlier samples does not drive the trends we see.

--Response:
We enhanced the quality of the figures using vector graphics and submitted them separately and in the manuscript. We hope
conversion will maintain the quality of the figures for your review. If accepted, we will address any concerns regarding the fig
the prosecution team. Please also refer to the --Responses to your revisions below.

Next, I will detail some major the shortcomings in the analysis I found in the figures:

1. The authors claim histone marks from six tissues and ATAC-seq from three tissues. However, from the Methods section, it
exactly the authors performed ChromHMM annotation. Did they build a model from the available data and then annotate the
they make a separate model for each tissue? I couldn't verify this since the authors didn't provide the code. How did the auth
the tissues that missed the ATAC-seq in the former case? Does that mean that the ATAC-seq presence is imputed from the hi
How did the authors verify and validate that the annotations are correct despite missing the crucial assay? This issue needs t
in the text, and the authors need to be forthcoming about this.

--Response:
We added the code to GitHub for your revisions. ChromHMM used the provided ChIP-seq data from 6 tissues and ATAC-seq d

tissues to build a model. https://github.com/rafet2005/Functional-annotation-of-regulatory-elements-in-rainbow-trout

2. One of the links to the UCSC browser session in Data Availability doesn't work. In the genome browser link provided, there
ChromHMM annotations, and I cannot see the data from which Figure 1A was generated. The authors should provide the indiv
from MACS and the assay signals (e.g. fold change of coverage over input control) in Bigwig format to show the intensities ar
enrichment.

--Response:
Again, sorry that the link did not work, we made sure it is working:
https://genome.ucsc.edu/cgi-bin/hgTracks?hgS_doOtherUser=submit&hgS_otherUserName=Rafet&hgS_otherUserSessionName=GCF_013265735.2%2FG

3. Figure 1B lacks the emission probabilities labels, making it difficult to interpret. The authors should provide clear labelling,
significantly improve the straightforward interpretation of the data. Additionally, Figure 1G should be presented as a table for

--Response:
Figure 1B was amended to have the emission probabilities. Figure 1G is now presented as a table.

4. I have some issues with naming chromatin states: genic enhancers are not enriched in the gene body, so I don't understan
were called like that. For some elements called genic enhancers, I would expect to either be present in the gene body or have
chromatin mark, which marks active transcription but is not a part of the author's dataset. Moreover, Poised enhancers look r
background state with weak probabilities of some signal, with a size much broader than one would expect from a typical regu
Once again, it would be helpful to see the raw signal in these areas to estimate if it's indeed a regulatory element or a backgr

--Response:
After adding the chromatin emission probabilities, you can see that the genic enhancers are characterized by very strong ope
signal (ATAC-seq) and moderate H3K27ac and H3K4me1 signals. The fold enrichment table 1F, we think, shows that genic en
actually enriched in the gene body, perhaps slightly more toward TES and UTR than the TSS, and is also enriched in the expr
(0.37) than the repressed genes (0.05). Regarding the Poised enhancers, it's worth noting that the signals are weak. Howeve
identified the combinations of these signals as an independent chromatin state. The decision of whether to retain or discard t
challenging one. We opted to include a clarifying statement: " It is important to note that the EnhPois showed minimal chrom
modification and openness signals, yet ChromHMM identified them as a chromatin state."

5. Figure 1H is a screenshot from the UCSC browser with labels put on top either in MS Word or MS PowerPoint. The authors
using the UCSC browser's possibilities to export publication-ready figures. Moreover, the labels on the figure are unreadable,
name and orientation are missing. The authors should also consider another example of the gene where the Active TSS doesn
entire gene but is instead localised in the promoter region.

--Response:
A new figure for the MLC1 was generated as instructed, thank you.

6. Finally, in Figure 1H, I find the enrichment of CpG methylation rather unexpected. The authors should check to ensure they
the chromatin states. I find it unbelievable that the Quiescent state, with a median length of 87kb and representing the part o
without any signal (practically random genomic regions), would have such a sharp methylation peak precisely in the middle. T
would better fit the Polycomb Repressed state, which is present in the hypermethylated regions. A similar observation is for t
ATAC-CpG regions. The authors should re-check their labellings and support these observations with individual examples.

--Response:
This sharp peak in methylation is caused by plotting the percentage of CpG methylation relative to the middle of each state. W
midpoint of each state as an arbitrary point to generate the figures, and as the states you referred to are relatively long, the
sharp in the middle. To avoid confusion, we removed the graphs of the long states 6-10, keeping the active states (1-5) figur

7. In Chromatin state correlation, the authors described the method of determining tissue-specific genes. However, they didn
definition and threshold for silenced genes as opposed to those that were not expressed.

--Response:
In our chromatin mark correlation analysis, we defined tissue-specific genes as those predominantly expressed in specific tiss

to other tissues where they are silent or scarce. We used "silenced" and "not expressed" interchangeably. To ensure consiste
"not expressed" with "silenced." We also included the following statement in the Methods section to clarify further: "The same
considered silenced in the other tissues, indicating no or almost no expression, for comparison."

8. Figure 2 should benefit from some statistical testing. For example, are the trends observed in Figures 2A, B, C, and D stati
significant when compared with a random set of promoters?

--Response:
We added statistical significance values (highlighted) to the text describing the figure.

9. In Figure 3, the original ROSE algorithm for defining super-enhancers removes the elements at a certain distance from the
promoter biases when calling super-enhancers. Considering the super-enhancers the authors represented are primarily enrich
promoter region, I wonder if the authors have considered this.

--Response:
We used the default parameters to run HOMAR which uses the Young lab algorithms (ROSE) where this step is optional
http://younglab.wi.mit.edu/super_enhancer_code.html
Here is the instruction “TSS_EXCLUSION_ZONE_SIZE: exclude regions contained within +/- this distance from TSS in order t
promoter biases (Default: 0; recommended if used: 2500). If this value is 0, will not look for a gene file.”
Here is the link to HOMAR
http://homer.ucsd.edu/homer/ngs/peaks.html#Finding_Super_Enhancers

10. Figure 3F should be presented as a table or in some other form. The GO terms are broad. Can the authors give a specific
tissue-specific SE associated with tissue-specific genes?

--Response:
We converted figure 3F to a table and moved it to additional file 4. We provided an example and amended the text to read “F
muscle-specific SE at location NC_048582.1:54022672-54039559 was associated with the muscle-specific gene Guanosine M
Reductase (GMPR).”

11. Also, this figure would benefit from a browser screenshot showing an example of a higher H3K27ac signal in super enhan
a typical enhancer.

--Response:
We added a figure (Fig.3F) as recommended.

12. Figure 4A is missing statistics. Are these overlaps statistically significant? Please provide these numbers as a fold change
versus expected by chance.

--Response:
A new figure was generated as requested.

13. Figure 4B is not intuitive to me. What is supposed to be represented on the y-axis? Please describe how these plots shoul
and read.

--Response:
To simplify the data, we generated a new figure showing the number of SNAs and Genes in QTL that overlapped with Enhanc
transcription factor binding sites.

14. The authors didn't clearly state how they calculated fold enrichments in Figure 5. Did they use chromatin mark peaks in F

--Response:
The calculation of fold enrichment was performed as described in the highlighted text:
“Sequencing reads were trimmed with Trim Galore (v.0.6.5)[56] and aligned with bowtie2[57] (v.2.5.4a) to the RBT genome
Accession GCA_013265735.3), and then duplicates were marked using Picard (v.2.18.7). MACS2 was used to call regions of s
enrichment ("peaks") [58].”
In text we detailed how fold enrichment ratios were log2-transformed and then converted to z scores in order to evaluate the

divergence of histone modification patterns among duplicate gene pairs.
“We first calculated the log2-transformed fold enrichment ratio. Then, we converted these ratios into z scores using the formu [illegible]
μ)/δ as in [63]. In this equation, χ represents the ratio value for a specific gene, μ denotes the mean ratio of all genes, and δ [illegible]
standard deviation of this ratio across all genes.
To assess the correlation and divergence of histone modification patterns between duplicate gene pairs, we utilized the Pears [illegible]
coefficient “r” of the histone modification profiles for the duplicated gene pair and dissimilarity index (1-r), respectively. By co [illegible]
mean values of “r” or “1-r” in each gene category, we determined the significance using the Wilcoxon rank-sum test.”

15. Why didn't they do it for each state separately in Figure 5B?

--Response:
Per recommendation from reviewer 2, we added table 3, describing the relative enrichment of the chromatin states in the sin [illegible]
compared to the conserved genes, neofunctionalized and specialized gene.

16. The authors didn't explain how they obtained the panels in Figure 5 in the Methods section. The authors must define this [illegible]
and make the code available.

--Response:
No custom code was used to conduct the analyses. All calculations were performed in Microsoft Excel. Please refer to the met [illegible]
titled "Divergence of Histone Modifications" for further information:
“We first calculated the log2-transformed fold enrichment ratio. Then, we converted these ratios into z scores using the formu [illegible]
μ)/δ as in [63]. In this equation, χ represents the ratio value for a specific gene, μ denotes the mean ratio of all genes, and δ [illegible]
standard deviation of this ratio across all genes.
To assess the correlation and divergence of histone modification patterns between duplicate gene pairs, we utilized the Pears [illegible]
coefficient “r” of the histone modification profiles for the duplicated gene pair and dissimilarity index (1-r), respectively. By co [illegible]
mean values of “r” or “1-r” in each gene category, we determined the significance using the Wilcoxon rank-sum test.”

17. Also, each bar plot should show the number of gene pairs tested. The interquartile widths of all the bar plots look suspicio [illegible]
considering the authors claim to have identified around 8,000 conserved, approximately 1500 neofunctionalised, and approxi [illegible]
specialised gene pairs.

--Response:
We added the number of tested genes into the figures.

In addition, I also have some minor comments:
Line 34 - Please introduce RBT as an acronym since it is the first sentence of the Introduction

--Response:
Done, thank you.

Line 39 is maybe a bit of an overstatement. There is more biological knowledge for other fish species, such as popular model [illegible]
zebrafish and medaka.

--Response:
Tuned down as suggested.
Line 42 - 49 references should be added.

--Response:
A reference was added.

Line 94 - As a part of the FAANG project, the authors should probably be aware of and look into the AQUA-FAANG project (https://www.aqua-faang.eu/)

--Response:
We added “In the EU, the AQUA-FAANG project aims to provide functional annotation tracks of six aquaculture species [30]. [illegible]
hand, in the US, over the last ten years of the FAANG project, aquaculture was represented by one species, the RBT. As part [illegible]
consortium, the main aim of this study was to annotate the RBT”

Line 96 - I believe the authors refer to the FAANG consortium here. Please make it clear.

--Response:
Yes, corrected.

Line 388 and 392 - it's promoters, not promotors.

--Response:
Corrected.

Line 388 and 392 - Promoters and transcripts are not the same or interchangeable as the authors suggest with promoters/tra
possible to claim to have identified transcripts without evidence of transcription.

--Response:
"Transcripts" removed.

Line 390 - 401 should be summarised and added as a Figure. If another similar dataset is available in the meantime, the autl
include it in their results and QC.

--Response:
The Ensemble annotations include promoters and enhancers and are not easily comparable to the 10 states reported in this s

Reviewer #2:
This submission described rainbow trout epigenome annotation combing ChIP-seq, ATAC-seq,Methyl Mini-seq, and RNA-seq c
related sequencing data process and related bioinformatics are comprehensive and adequate. The epigenome annotation map
associated analysis and results make sense. In my opinion, Annotating the genome resources are valuable resource for the d
regulatory elements, functional research, and other prospective investigations. I have some concerns that should be addresse
accepted.

1. The present study appears to be a data description work or a database paper. Is it feasible to conduct comparative analysi
different tissues? What about the available data for each tissue? Are there distinct patterns observed in ChIP-seq, ATAC-seq,
even Methyl Mini-seq across different samples?

Response:
Dear Dr. Jian,
Thank you for reviewing our MS. The study characterized the tissue specificity of histone states and their relationship with tis
gene expression (Pages 196-228, Section "HISTONE MODIFICATION ASSOCIATION WITH GENE EXPRESSION"). Additionally,
SUPER-ENHANCERS in each tissue was characterized (Pages 254-266).

2. The reference genome serves as a valuable resource for mapping, gene discovery, and marker development, whereas obta
epigenome information from reference annotation appears to be more challenging. Is it possible to generate description files
file or any other file formats?

--Response:
A chromatin description file (Supplementary file 7) was added.

3. The authors have highlighted the unique potential of RBT as a model organism for studying the early stages of gene evolut
recent salmonid-specific whole genome duplication and the presence of large duplicated regions. It is worth investigating whe
duplicate regions exhibit distinct patterns in comparison to other genomic regions, particularly in terms of epigenome annotat
salmonid-specific WGD, the RBT become a diploid again. Maybe it is also a challenge for us to define clearly the duplicated ge
singleton. So I think the methods should be more carefully in the analysis.

--Response:
We carefully described the methods of identifying duplicate genes under the subtitle "Identification of Genes in Collinear Bloc
updated the description of the methods used for " Divergence of Histone Modifications." Per your suggestion, we added table

the relative enrichment of the chromatin states in the single-copy genes compared to the conserved genes, neofunctionalized
specialized genes. This was a good addition,
Thank you.
