# Supplementary material for: Functional annotation of regulatory elements in rainbow trout uncovers roles of the epigenome in genetic selection and genome evolution
Source: Gigascience. 2024 Dec 4;13:giae092. doi: 10.1093/gigascience/giae092 (PMC11629980; doi:10.1093/gigascience/giae092)
Supplement: giae092_Response_to_Reviewer_Comments_Revision_1 [file giae092_response_to_reviewer_comments_revision_1.pdf]

Author's Response To Reviewer Comments

Please see the attached "Response to Reviewer Comments" file on the last pages below. We attached the file to maintain the enclosed table formats.

Thank you,
