# Supplementary material for: Functional annotation of regulatory elements in rainbow trout uncovers roles of the epigenome in genetic selection and genome evolution
Source: Gigascience. 2024 Dec 4;13:giae092. doi: 10.1093/gigascience/giae092 (PMC11629980; doi:10.1093/gigascience/giae092)
Supplement: giae092_Reviewer_1_Report_Original_Submission — Damir BaranaÅ¡iÄ‡ -- 5/26/2024 [file giae092_reviewer_1_report_original_submission.pdf]

Reviewer Report

Title: Functional annotation of regulatory elements in rainbow trout uncovers roles of the epigenome in genetic selection and genome evolution

Version: Original Submission **Date:** 5/26/2024

Reviewer name: Damir BaranaÅ¡iÄ‡

Reviewer Comments to Author:

The manuscript titled "Functional annotation of regulatory elements in rainbow trout uncovers roles of the epigenome in genetic selection and genome evolution" from Salem et al. provides an atlas of regulatory elements in six tissues of aquaculturally important rainbow trout species. Although the authors provided a notable amount of novel data, I must raise major concerns regarding the data processing and downstream data analysis. My impression is that the analyses were often done without proper quality control or deeper consideration and literature consultation, and most of the analyses should be rerun with some additional criteria in mind. I will address most of the issues below. I would like to emphasise three main problems which recur throughout the manuscript:
a. The quality control of the raw and processed data is missing. It is hard to assess the quality of the analyses and annotations without considering how good the raw data are. The quality control should include at least a fraction of reads in peaks and some PCA plots showing the data separate by tissue and reproducibility. Also, the authors should provide statistics on how well the peaks overlap between replicates. In addition, the peaks and the signal tracks must be available in the genome browser, along with all the annotations produced in the manuscript.
b. The code for the analyses is not available. The code to reproduce the analyses in the manuscript should be publicly available on GitHub or some other public code repository.
c. It's crucial to emphasise that the Figure quality is currently low. Some screenshots have low-resolution labels that are hard to read. The authors need to provide the figures as vector graphics of sufficient quality, as this will significantly enhance the clarity and impact of their research. Additionally, since the manuscript includes many profile plots and meta plots, adding a significance interval of the signals would be helpful to ensure that a small number of outlier samples does not drive the trends we see.
Next, I will detail some major the shortcomings in the analysis I found in the figures:
1. The authors claim histone marks from six tissues and ATAC-seq from three tissues. However, from the Methods section, it is unclear how exactly the authors performed ChromHMM annotation. Did they build a model from the available data and then annotate the genome, or did they make a separate model for each tissue? I couldn't verify this since the authors didn't provide the code. How did the authors deal with the tissues that missed the ATAC-seq in the former case? Does that mean that the ATAC-seq presence is imputed from the histone marks? How did the authors verify and validate that the annotations are correct despite missing the crucial assay? This issue needs to be addressed in the text, and the authors need to be forthcoming about this.
2. One of the links to the UCSC browser session in Data Availability doesn't work. In the genome browser link provided, there are only ChromHMM annotations, and I cannot see the data from which Figure 1A

was generated. The authors should provide the individual peaks from MACS and the assay signals (e.g. fold change of coverage over input control) in Bigwig format to show the intensities and local enrichment.

3. Figure 1B lacks the emission probabilities labels, making it difficult to interpret. The authors should provide clear labelling, as this will significantly improve the straightforward interpretation of the data. Additionally, Figure 1G should be presented as a table for better clarity.

4. I have some issues with naming chromatin states: genic enhancers are not enriched in the gene body, so I don't understand why they were called like that. For some elements called genic enhancers, I would expect to either be present in the gene body or have the H3K36me3 chromatin mark, which marks active transcription but is not a part of the author's dataset. Moreover, Poised enhancers look more like a background state with weak probabilities of some signal, with a size much broader than one would expect from a typical regulatory element. Once again, it would be helpful to see the raw signal in these areas to estimate if it's indeed a regulatory element or a background state.

5. Figure 1H is a screenshot from the UCSC browser with labels put on top either in MS Word or MS PowerPoint. The authors should consider using the UCSC browser's possibilities to export publication-ready figures. Moreover, the labels on the figure are unreadable, and the gene name and orientation are missing. The authors should also consider another example of the gene where the Active TSS doesn't cover the entire gene but is instead localised in the promoter region.

6. Finally, in Figure 1H, I find the enrichment of CpG methylation rather unexpected. The authors should check to ensure they didn't mislabel the chromatin states. I find it unbelievable that the Quiescent state, with a median length of 87kb and representing the part of the genome without any signal (practically random genomic regions), would have such a sharp methylation peak precisely in the middle. This profile would better fit the Polycomb Repressed state, which is present in the hypermethylated regions. A similar observation is for the EnhPois and ATAC-CpG regions. The authors should re-check their labellings and support these observations with individual examples.

7. In Chromatin state correlation, the authors described the method of determining tissue-specific genes. However, they didn't mention the definition and threshold for silenced genes as opposed to those that were not expressed.

8. Figure 2 should benefit from some statistical testing. For example, are the trends observed in Figures 2A, B, C, and D statistically significant when compared with a random set of promoters?

9. In Figure 3, the original ROSE algorithm for defining super-enhancers removes the elements at a certain distance from the TSS to remove promoter biases when calling super-enhancers. Considering the super-enhancers the authors represented are primarily enriched in the promoter region, I wonder if the authors have considered this.

10. Figure 3F should be presented as a table or in some other form. The GO terms are broad. Can the authors give a specific example of tissue-specific SE associated with tissue-specific genes?

11. Also, this figure would benefit from a browser screenshot showing an example of a higher H3K27ac signal in super enhancer compared to a typical enhancer.

12. Figure 4A is missing statistics. Are these overlaps statistically significant? Please provide these numbers as a fold change of observed versus expected by chance.

13. Figure 4B is not intuitive to me. What is supposed to be represented on the y-axis? Please describe how these plots should be interpreted and read.

14. The authors didn't clearly state how they calculated fold enrichments in Figure 5. Did they use chromatin mark peaks in Figure 5A?
15. Why didn't they do it for each state separately in Figure 5B?
16. The authors didn't explain how they obtained the panels in Figure 5 in the Methods section. The authors must define this method in detail and make the code available.
17. Also, each bar plot should show the number of gene pairs tested. The interquartile widths of all the bar plots look suspiciously narrow, considering the authors claim to have identified around 8,000 conserved, approximately 1500 neofunctionalised, and approximately 1,300 specialised gene pairs.

In addition, I also have some minor comments:

Line 34 - Please introduce RBT as an acronym since it is the first sentence of the Introduction

Line 39 is maybe a bit of an overstatement. There is more biological knowledge for other fish species, such as popular model organisms, zebrafish and medaka.

Line 42 - 49 references should be added.

Line 94 - As a part of the FAANG project, the authors should probably be aware of and look into the AQUA-FAANG project (https://www.aqua-faang.eu/)

Line 96 - I believe the authors refer to the FAANG consortium here. Please make it clear.

Line 388 and 392 - it's promoters, not promotors.

Line 388 and 392 - Promoters and transcripts are not the same or interchangeable as the authors suggest with promoters/transcript. It is not possible to claim to have identified transcripts without evidence of transcription.

Line 390 - 401 should be summarised and added as a Figure. If another similar dataset is available in the meantime, the authors should include it in their results and QC.

Methods

Are the methods appropriate to the aims of the study, are they well described, and are necessary controls included? Choose an item.

Conclusions

Are the conclusions adequately supported by the data shown? Choose an item.

Reporting Standards

Does the manuscript adhere to the journal's guidelines on minimum standards of reporting? Choose an item.

Choose an item.

Statistics

Are you able to assess all statistics in the manuscript, including the appropriateness of statistical tests used? Choose an item.

Quality of Written English

Please indicate the quality of language in the manuscript: Choose an item.

Declaration of Competing Interests

Please complete a declaration of competing interests, considering the following questions:

- Have you in the past five years received reimbursements, fees, funding, or salary from an organisation that may in any way gain or lose financially from the publication of this manuscript, either now or in the future?
- Do you hold any stocks or shares in an organisation that may in any way gain or lose financially from the publication of this manuscript, either now or in the future?
- Do you hold or are you currently applying for any patents relating to the content of the manuscript?
- Have you received reimbursements, fees, funding, or salary from an organization that holds or has applied for patents relating to the content of the manuscript?
- Do you have any other financial competing interests?
- Do you have any non-financial competing interests in relation to this paper?

If you can answer no to all of the above, write 'I declare that I have no competing interests' below. If your reply is yes to any, please give details below.

I was part of the AQUA-FAANG consortium, and part of my analyses include the use of functional annotations of adult tissues in rainbow trout. However, the authors results are in no conflict with my own analyses and this manuscript don't represent a competition to my analyses.

I agree to the open peer review policy of the journal. I understand that my name will be included on my report to the authors and, if the manuscript is accepted for publication, my named report including any attachments I upload will be posted on the website along with the authors' responses. I agree for my report to be made available under an Open Access Creative Commons CC-BY license (http://creativecommons.org/licenses/by/4.0/). I understand that any comments which I do not wish to be included in my named report can be included as confidential comments to the editors, which will not be published.

Choose an item.

To further support our reviewers, we have joined with Publons, where you can gain additional credit to further highlight your hard work (see: https://publons.com/journal/530/gigascience). On publication of this paper, your review will be automatically added to Publons, you can then choose whether or not to claim your Publons credit. I understand this statement.

Yes Choose an item.
