# Supplementary material for: Functional annotation of regulatory elements in rainbow trout uncovers roles of the epigenome in genetic selection and genome evolution
Source: Gigascience. 2024 Dec 4;13:giae092. doi: 10.1093/gigascience/giae092 (PMC11629980; doi:10.1093/gigascience/giae092)
Supplement: giae092_Reviewer_2_Report_Original_Submission — Jianbo Jian -- 6/17/2024 [file giae092_reviewer_2_report_original_submission.pdf]

Reviewer Report

Title: Functional annotation of regulatory elements in rainbow trout uncovers roles of the epigenome in genetic selection and genome evolution

Version: Original Submission **Date:** 6/17/2024

Reviewer name: Jianbo Jian

Reviewer Comments to Author:

This submission described rainbow trout epigenome annotation combing ChIP-seq, ATAC-seq,Methyl Mini-seq, and RNA-seq data. All the related sequencing data process and related bioinformatics are comprehensive and adequate. The epigenome annotation maps and associated analysis and results make sense. In my opinion, Annotating the genome resources are valuable resource for the discovery of regulatory elements, functional research, and other prospective investigations. I have some concerns that should be addressed before accepted.

1. The present study appears to be a data description work or a database paper. Is it feasible to conduct comparative analysis among different tissues? What about the available data for each tissue? Are there distinct patterns observed in ChIP-seq, ATAC-seq, RNA-Seq, or even Methyl Mini-seq across different samples?
2. The reference genome serves as a valuable resource for mapping, gene discovery, and marker development, whereas obtaining epigenome information from reference annotation appears to be more challenging. Is it possible to generate description files just like the gff file or any other file formats?
3. The authors have highlighted the unique potential of RBT as a model organism for studying the early stages of gene evolution, given its recent salmonid-specific whole genome duplication and the presence of large duplicated regions. It is worth investigating whether these duplicate regions exhibit distinct patterns in comparison to other genomic regions, particularly in terms of epigenome annotation. After salmonid-specific WGD, the RBT become a diploid again. Maybe it is also a challenge for us to define clearly the duplicated genes or singleton. So I think the methods should be more carefully in the analysis.

Methods

Are the methods appropriate to the aims of the study, are they well described, and are necessary controls included? Choose an item.

Conclusions

Are the conclusions adequately supported by the data shown? Choose an item.

Reporting Standards

Does the manuscript adhere to the journal's guidelines on [minimum standards of reporting?](#) Choose an item.

Choose an item.

Statistics

Are you able to assess all statistics in the manuscript, including the appropriateness of statistical tests used? Choose an item.

Quality of Written English

Please indicate the quality of language in the manuscript: Choose an item.

Declaration of Competing Interests

Please complete a declaration of competing interests, considering the following questions:

- Have you in the past five years received reimbursements, fees, funding, or salary from an organisation that may in any way gain or lose financially from the publication of this manuscript, either now or in the future?
- Do you hold any stocks or shares in an organisation that may in any way gain or lose financially from the publication of this manuscript, either now or in the future?
- Do you hold or are you currently applying for any patents relating to the content of the manuscript?
- Have you received reimbursements, fees, funding, or salary from an organization that holds or has applied for patents relating to the content of the manuscript?
- Do you have any other financial competing interests?
- Do you have any non-financial competing interests in relation to this paper?

If you can answer no to all of the above, write 'I declare that I have no competing interests' below. If your reply is yes to any, please give details below.

I declare that I have no competing interests

I agree to the open peer review policy of the journal. I understand that my name will be included on my report to the authors and, if the manuscript is accepted for publication, my named report including any attachments I upload will be posted on the website along with the authors' responses. I agree for my report to be made available under an Open Access Creative Commons CC-BY license (http://creativecommons.org/licenses/by/4.0/). I understand that any comments which I do not wish to be included in my named report can be included as confidential comments to the editors, which will not be published.

Choose an item.

To further support our reviewers, we have joined with Publons, where you can gain additional credit to further highlight your hard work (see: https://publons.com/journal/530/gigascience). On publication of this paper, your review will be automatically added to Publons, you can then choose whether or not to claim your Publons credit. I understand this statement.

Yes Choose an item.
